# Mobilizing community-driven health promotion through community granting programs: a rapid systematic review

**DOI:** 10.1186/s12889-024-18443-8

**Published:** 2024-04-01

**Authors:** Emily C. Clark, Shamara Baidoobonso, Karen A. M. Phillips, Laura Lee Noonan, Jiselle Bakker, Trish Burnett, Karlene Stoby, Maureen Dobbins

**Affiliations:** 1 National Collaborating Centre for Methods and Tools, McMaster University, McMaster Innovation Park, 175 Longwood Rd S, Suite 210a, Hamilton, ON L8P 0A1 Canada; 2 Department of Health and Wellness, Government of Prince Edward Island, Chief Public Health Office, 16 Fitzroy St, Charlottetown, PE C1A 7N8 Canada; 3 School of Nursing, McMaster University, Health Sciences Centre 2J20, 1280 Main St W, Hamilton, ON L8S 4K1 Canada

**Keywords:** Community grant program, Community mobilization, Community engagement, Academic research partnership, Health promotion, Public health, Health equity

## Abstract

**Background:**

Effective health promotion responds to the unique needs of communities. Community granting programs that fund community-driven health promotion initiatives are a potential mechanism to meet those unique needs. While numerous community health-focused programs are available, the various strategies used by granting programs to foster engagement, administer grants and support awardees have not been systematically evaluated. This rapid systematic review explores the administration of community granting programs and how various program components impact process and population health outcomes.

**Methods:**

A systematic search was conducted across three databases: Medline, SocINDEX, and Political Science Database. Single reviewers completed screening, consistent with a rapid review protocol. Studies describing or evaluating community granting programs for health or public health initiatives were included. Data regarding program characteristics were extracted and studies were evaluated for quality. A convergent integrated approach was used to analyze quantitative and qualitative findings.

**Results:**

Thirty-five community granting programs, described in 36 studies, were included. Most were descriptive reports or qualitative studies conducted in the USA. Program support for grant awardees included technical assistance, workshops and training, program websites, and networking facilitation. While most programs reported on process outcomes, few reported on community or health outcomes; such outcomes were positive when reported. Programs reported that many funded projects were likely sustainable beyond program funding, due to the development of awardee skills, new partnerships, and securing additional funding. From the perspectives of program staff and awardees, facilitators included the technical assistance and workshops provided by the programs, networking amongst awardees, and the involvement of community members. Barriers included short timelines to develop proposals and allocate funds.

**Conclusions:**

This review provides a comprehensive overview of health-related community granting programs. Grant awardees benefit from technical assistance, workshops, and networking with other awardees. Project sustainability is enhanced by the development of new community partnerships and grant-writing training for awardees. Community granting programs can be a valuable strategy to drive community health, with several key elements that enhance community mobilization.

**Registration:**

PROSPERO #CRD42023399364.

**Supplementary Information:**

The online version contains supplementary material available at 10.1186/s12889-024-18443-8.

## Background

Communities have unique health needs and priorities determined by, among other factors, population characteristics, built environments and social determinants of health [1, 2]. Public health is tasked with assessing the needs of the communities they serve and implementing programs, services, and policies that align with community priorities to prevent injury, illness, and premature death [3–6]. Understanding community context is a cornerstone of the evidence-informed approach to public health practice, where evidence from research and practice are integrated in decision-making [7, 8]. Health promotion is a critical function of public health and includes implementing interventions that enable individuals and communities to improve their health. For example, such programs can support healthy nutrition, physical activity, and mental wellness [9]. However, it can be challenging for public health to meet distinct health promotion needs of communities within the populations they serve; what works in one community may not be as effective in another [7, 10].

It has been suggested that the effectiveness of health promotion efforts may be improved by community-informed approaches that build on particular strengths and respond to needs of the community [11]. Community involvement in developing health promotion initiatives empowers community in driving their own health outcomes [12]. Where health behaviour changes require multiple and persistent influences to support sustained changes, community engagement can drive these influences [13]. While public health often engages community members in consultation for program and service development, community-driven initiatives are those that have been developed by the community, for the community [14]. While community-driven approaches have also been conceptualized as community-based health promotion, community-led programs, or community-based participatory research, the common thread is that change is initiated and driven by community members, rather than by government or academic bodies [14–17]. A recent systematic review of community-driven health promotion and disease prevention initiatives found promising results for urban community-driven interventions in improving health outcomes [17]. Another systematic review of community participation in health services demonstrated positive outcomes at community and individual levels [11]. Impacts were greatest for non-communicable disease health outcomes, such as physical activity and quality of life, which align well with health promotion activities [11]. For populations made vulnerable through structural inequities, a meta-analysis of public health interventions for a broad range of health topics found that community engagement was associated with significant effects for health behaviour outcomes, health behaviour self-efficacy and perceived social support [18].

Fostering community action by providing funding for community-driven health promotion initiatives is a potential mechanism to address unique local health needs [11, 19, 20]. There are numerous community health-focused granting programs available at local, regional, and national levels in Canada and beyond. For example, municipalities and regions offer grants to fund community-led projects that promote health and well-being [21, 22]. Many provinces and territories in Canada fund health, recreation, and culturally-focused community building grants [23–25]. There are also community health granting programs available through non-profit and for-profit organizations, as well as the federal government, for community-driven health initiatives. [26–32]. Community granting programs typically administer a pool of funds available to community-based organizations to implement projects. Often, grant applications from community-based organizations propose projects within a scope defined by the granting organization. The community granting program sometimes provides support to awardees, such as training to develop relevant skills and technical assistance consultations from program staff to support planning, implementation, or evaluation of projects. There are no set standards for administering a health promotion grant program. Examples of community granting programs in the literature vary in terms of application and reporting requirements, the supports available to applicants and awardees, and the reporting of program-level and project-level outcomes.

Community granting programs are well-suited for health promotion projects, as both focus on strengthening community action [4]. Small community grants for health promotion have been found to stimulate innovations and engage new community organizations [15]. While community grants for health promotion are prevalent in Canada and worldwide, there has not yet been a systematic review exploring how different components of granting programs affect their success. This paper takes a rapid systematic review approach to address this gap, in order to inform the development of a community granting program in a Canadian province within a discrete timeline. Rapid reviews allow for the production of evidence syntheses within a shorter timeframe, allowing for timely access to synthesized evidence [33]. While there are methodological limitations to a rapid approach to reviews, various efforts can minimize these limitations [34]. This rapid systematic review explores: 1. how community granting programs have been administered, and 2. which components are associated with success, both in terms of process outcomes and achieving population health outcomes. Specifically, this review includes papers that describe or evaluate the granting programs themselves, rather than the projects that they funded. This review will inform the design and implementation of health-focused community granting programs that mobilize community-based organizations in addressing the unique health needs of their communities.

## Methods

### Study design

This review was completed by the National Collaborating Centre for Methods and Tools’ Rapid Evidence Service [35, 36]. The review was conducted and reported following the Preferred Reporting Items for Systematic Reviews and Meta-Analyses (PRISMA) statement for reporting systematic reviews and meta-analyses [37]. The review protocol was registered with the International Prospective Register of Systematic Reviews (PROSPERO; Registration CRD42023399364).

### Information sources and search strategy

A health librarian supported search strategy development and conducted the search on March 16, 2023. The following three databases were searched from inception: Medline, SocINDEX, and Political Science Database. Databases were searched using combinations of terms related to “grant”, “subsidy”, “endowment”, “financing” and “community”. The full search strategy is included in Appendix 1.

DistillerSR software was used to screen articles. Two reviewers screened a subset of 100 articles at the title and abstract level, achieving over 90% agreement. A single reviewer screened the remaining titles and abstracts of retrieved studies. A second reviewer screened full texts of included studies. Duplicate screening was not used for the entire reference set, consistent with a rapid review protocol [36].

### Eligibility criteria

English-language primary studies with either experimental or observational designs were eligible for inclusion. Syntheses, such as literature and systematic reviews, were excluded. Eligibility criteria are reported in accordance with a PICOS (Population, Intervention, Comparator, Outcomes, Setting) question framework [38].

#### Population

Studies of granting programs available to communities and non-profit community groups were included. Communities were broadly defined as social groups that have a common trait, such as their location of residence, culture or faith, or institution (such as a school or workplace). Community groups eligible for grants included youth-serving organizations, non-government organizations, business communities or municipalities. Grant programs for professional groups, consumers, labour unions, researchers or research consortia were excluded.

#### Intervention

Community granting programs for projects related to health or public health topic areas were included, such as health promotion, the structural determinants of health or environmental health. Studies of programs with total annual budgets of greater than $500 000 CAD were excluded to allow application of this review’s findings to the development of a smaller-scale community granting program. Crowd funding initiatives were excluded.

Granting programs linked to research funding were included when the project funding was awarded to paired researchers and community partners to implement community-driven participatory research projects. For inclusion in this review, project proposals must have been developed in partnership with community-based organizations or individuals.

#### Comparator

Given the nature of the intervention, studies were not required to have included a comparator for inclusion. Qualitative studies and descriptive case reports were eligible for inclusion in this review.

#### Outcomes

Outcomes that were measured either qualitatively or quantitatively were included. Quantitative outcomes included the number and types of projects proposed or implemented, as well as community-level or population-level outcomes. Given the expected heterogeneity in study designs and reported outcomes, any type of community or population-level outcomes were eligible for this review. This includes health behaviour outcomes, e.g., reports of physical activity or diet; population levels of health-related screening, e.g., for cancer or sexually transmitted infections; changes to the built environment, e.g., development of green space; or reports of community knowledge, e.g., for health-related topics. Qualitative findings on lessons learned, facilitators and barriers for community granting programs were included.

#### Setting

Studies conducted in low- and middle-income countries were excluded to allow application of this review’s findings to the development of a community granting program in Canada, [39].

### Quality Assessment

The Joanna Briggs Institute (JBI) suite of critical appraisal tools was used to evaluate the quality of included studies [40]. Single-group pre-post studies were assessed using the JBI Checklist for Quasi-Experimental Studies. Qualitative and cross-sectional studies were assessed using their corresponding JBI checklists. Studies were rated low, moderate, or high quality according to critical appraisal results. Two reviewers completed quality assessment independently and conflicts were resolved through discussion. Descriptive studies provided an overview of a granting program or its implementation, reporting on some outcomes and the authors’ reflections on the program. Since these studies did not conduct a formal analysis or program evaluation they were not appraised for methodological quality.

### Data extraction

Data extraction was completed by a single reviewer and verified by a second reviewer. Data on the study design, location, grant size, granting organization, eligible projects and recipients, program components, and outcomes were extracted when reported.

### Data analysis

A convergent integrated approach was used to synthesize quantitative and qualitative data simultaneously [41]. Common granting program elements were extracted and summarized, including grant application processes, application review and selection processes, reporting requirements, technical assistance provided by the granting program, and project sustainability. Qualitative findings were reviewed for commonalities and differences. Concepts were grouped and summarized by common themes [42].

Due to the heterogeneity in study outcomes and descriptive nature of many included studies, the Grading of Recommendations, Assessment, Development and Evaluations (GRADE) [43] approach was not applied to the findings of this review.

## Results

**Fig. 1:**
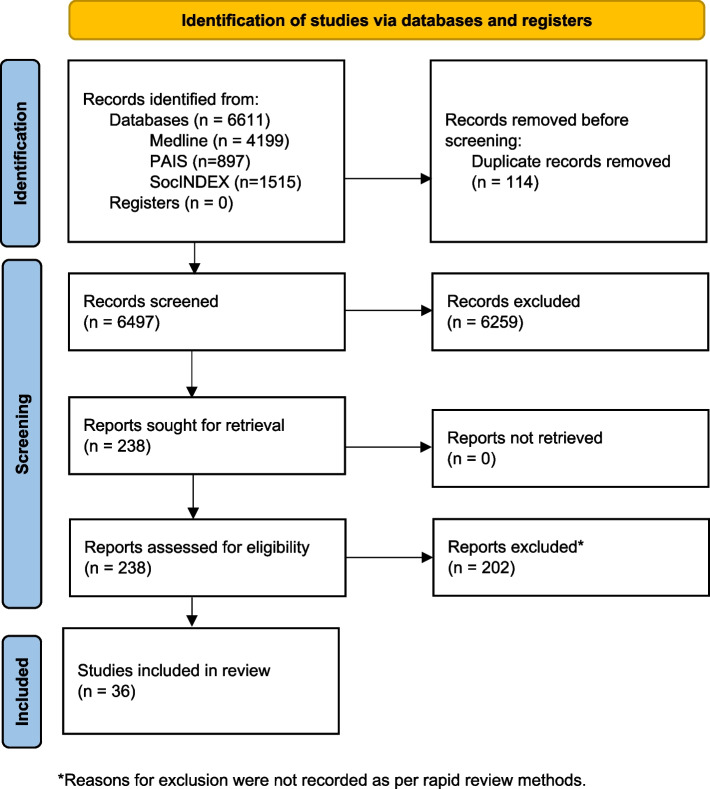
PRISMA 2020 Flow chart

A total of 6611 records were retrieved after database searching. Following the removal of duplicates, 6497 records were screened by title and abstract, of which 6259 were assessed as not relevant. The remaining 238 reports were reviewed at the full text level, of which 36 articles were included. A PRISMA flow chart illustrating the article search and selection process is included in Fig. 1. While the reason for study exclusion at the full text level was not recorded for all studies, consistent with a rapid review methodology, there were 22 studies of community grant programs that were excluded because the total funding pool of the program was greater than $500 000 CAD. Other studies were excluded because they focused on an evaluation of funded projects, rather than the granting program, or because they focused on community initiatives that were not funded by a granting program.

### Study Characteristics

There were two included articles that explored the same community granting program during separate time periods [44]. The description of the program and findings from these studies have been merged and considered as a single study for the purposes of this review. Nineteen studies that provided a descriptive overview of a granting program and its implementation, without a formal analysis or program evaluation, were labelled as descriptive studies and not appraised for quality. The overall characteristics of included studies are summarized in Table 1.

**Table 1:** Included studies of community granting programs

Study	Grant program, organization, location, grant size, framework	Focus area, eligible projects, eligible grant recipients	Granting program administration	Granting program components	Outcomes	Sustainability	Study design, quality rating
Abildso, 2019 [45]	**Program**: The Growing Healthy Community (GHC) Collaborative Grant Program **Organization**: Claude Worthington Benedum Foundation and the West Virginia Department of Health and Human Resources **Location**: West Virginia, USA **Grant size**: Max. $25 000 USD **Framework**: None	**Focus area**: Health promotion **Eligible projects**: Projects that provide access to healthy food e.g., community gardens, indoor farmers market, and spaces for physical activity, e.g., walking program, downtown wellness kiosk, often according to The Community Guide to Preventive Services Creating or Improving Places for Physical Activity or the Centers for Disease Control and Prevention’s Recommended Community Strategies and Measurements to Prevent Obesity in the United States **Eligible recipients**: Community organizations recognized by state economic development programs (Main Street West Virginia and West Virginia Organization, Training, Revitalization, and Capacity	Not described	Not described	38 projects funded across 24 communities Limited time to spend funds was a barrier Centralized resources and technical assistance recommended Program led to social cohesion within community and increased activity at local businesses	Several project leaders secured additional funding to sustain projects	**Study design**: Qualitative **Quality rating**: High
Alexander, 2020 [46]	**Program**: Meharry-Vanderbilt Community Engaged Research Core mini grant program **Organization**: Meharry-Vanderbilt Community Engaged Research Core (CERC) **Location**: USA **Grant size**: Max. $10 000 USD **Framework**: Patient Centered Outcomes Research Institute (PCORI) Principles of Community Engagement	**Focus area**: Public health (general) **Eligible projects**: Projects that address community-identified needs; examples not provided **Eligible recipients**: Community-based organizations, in partnership with academic researchers and/or graduate students	**Dissemination**: Calls for applications circulated biannually to community-based organizations **Application**: Potential applicants submit a letter of intent, then attend an information session. Applications submitted via an online web application. Application required a statement of purpose, potential impact, partner roles, anticipated outcomes, timeline, budget justification and research and dissemination plan. Applications were reviewed by committee of faculty and community members **Reporting**: Awardees required to submit mid- and end-of-project reports, share results at a community meeting	Not described	56 projects funded 2008–2018 In response to participant feedback: • Review committee expanded to include members of different races, • Application form standardized by adapting National Institutes of Health Research Grant Evaluation Rubric and review criteria, • Feedback was provided to applicants on applications that were not funded Program increased skills for awardees, such as evaluation, funding acquisition	Nearly 20 projects resulted in ongoing research partnerships. Preliminary data from granted programs strengthen subsequent applications for additional funds	**Study design**: Descriptive **Quality rating**: Not appraised
Allen, 2017 [47]	**Program**: Community Health Innovation Awards (CHIA) **Organization**: University of Alabama at Birmingham (UAB) **Location**: Birmingham, Alabama, USA **Grant size**: Max. $25 000 USD **Framework**: Community-based participatory research (CBPR) framework	**Focus area**: Public health (general) **Eligible projects**: Program conducted a survey of community members to identify a list of 12 neighbourhood concerns that could be addressed by proposed projects **Eligible recipients**: Neighborhood associations and non-profit organizations	**Dissemination**: Calls for application circulated through mail and organization’s affiliated websites **Application**: Applicants first submitted a draft proposal. Applicants with strong draft proposals invited to submit final proposal and deliver 10-min presentation to review committee. Committee scored applications using a customized rubric **Reporting**: Not described	**Technical Assistance**: Program mentors assigned to applicants guided application development **Training**: Awardees required to attend 3 workshops on innovative thinking, idea development, grant writing and application process	78 proposals received, and 26 projects funded 2012–2017 Key lessons learned include: • Engage communities at outset of program development, • Foster inclusive and participatory environments	Not described	**Study design**: Descriptive **Quality rating**: Not appraised
Baril, 2011 [48]	**Program**: No formal name **Organization**: Boston Public Health Commission’s Center for Health Equity and Social Justice **Location**: Massachusetts, Vermont, Connecticut, Rhode Island, and New Hampshire, USA **Grant size**: $25–30 000 USD annually for 3 years Framework: Boston Public Health Commission’s health equity framework and theory of change	**Focus area**: Social determinants of health **Eligible projects**: Projects that address social determinants of health, e.g., improving food environments, employment opportunities in health for youth of colour **Eligible recipients**: community-based organizations, educational institutions, community health centres, hospitals, neighbourhood associations, faith-based organizations, public health departments	**Dissemination**: Not described **Application**: Required a comprehensive project plan. Applicants were assessed for history of working with communities of colour, commitment to reducing health inequities and capacity for systems-level change **Reporting**: After year 1, required to submit strategic work plan of goals, activities and outputs. During years 2 and 3, required to report progress on objectives and complete Partnership Assessment Tool	**Technical Assistance**: Regular teleconferences between awardees and expert advisors, and among awardees to share learning. Program staff issued bimonthly email updates. Faculty consultants available to support coalition building, strategic planning, and promotion of antiracist social change **Training**: During year 1, awardees provided training on health equity framework, data collection and analysis for health equity, anti-racism. Optional training provided on coalition building, community organizing, community needs and asset assessments, policy advocacy, logical models and evaluation, and framing and communicating racial equity	15 projects funded 2008–2012 Outcomes not available at time of writing	Not described	**Study design**: Descriptive **Quality rating**: Not appraised
Bounds, 2011 [49]	**Program**: Community Cancer Control in Appalachia Forum **Organization**: National Comprehensive Cancer Control Program **Location**: Appalachian regions and Tennessee, USA **Grant size**: $2500 USD for roundtables or $5000 USD for forums **Framework**: Coalition theory	**Focus area**: Cancer prevention **Eligible projects**: Roundtables focused on local cancer risk, incidence, and death rates and introduction of state cancer plans or in-depth forums focused on cancer data, state cancer plans and successful cancer control programs in local communities **Eligible recipients**: Community organizations, state or regional cancer coalitions	**Dissemination**: Call for applications distributed through partner organizations **Application**: Description of the proposed event, including agenda, partners, plan to recruit speakers, budget justification, anticipated outcomes using a Give-Get Grid. Applications reviewed by program staff using guidelines approved by partner organizations **Reporting**: Final report required	Not described	9 forums and 19 roundtables funded Short deadline for applications resulted in few applications. The deadline was extended Program facilitated identification of local partners for cancer coalitions	Some coalitions obtained additional funding to conduct further forums	**Study design**: Descriptive **Quality rating**: Not appraised
Camponeschi, 2017 [50]	**Program**: No formal name **Organization**: Environmental Public Health Tracking Network (EPHTN) **Location**: Wisconsin, USA **Grant size**: Max. $10 500 USD **Framework**: Social Ecological Model of Health	**Focus area**: Environmental health **Eligible projects**: Any environmental health community projects informed by data from the EPHTN’s data portal **Eligible recipients**: Local and tribal health departments	**Dissemination**: Funding opportunity announcement issued to local and tribal health departments **Application**: Multiple EPHTN staff members scored applications according to a rubric: identified environmental health issue for target jurisdiction, well-defined project, goals, timeline, work plan, appropriate partners, evaluation plan and budget **Reporting**: Mid-project and final reports documenting successes, results and lessons learned	**Technical Assistance**: Program staff were assigned to each funded project to act as program liaisons. Awardees were offered assistance with materials development, connections to experts, guidance for evaluation planning, and developing a journal manuscript data collection and interpretation **Networking facilitation**: Conference calls were held together for awardees with similar projects	15 proposals received, and 8 projects funded in 9-month period. Staff provided estimated 10–15 h of technical assistance per project Awardees found technical assistance useful and had minimal suggestions for improving the program Awardees reported positive public health outcomes resulting from funded projects. Health department communication with communities was strengthened	Not described	**Study design**: Descriptive **Quality rating**: Not appraised
Caperchione, 2010 [51]	**Program**: Women's Active Living Kits (WALK) Community Grant Scheme **Organization**: Australian Office for Women, Department of Families, Community Services and Indigenous Affairs **Location**: Australian Capital Territory, Victoria, New South Wales and Queensland, Australia **Grant size**: Max. $1500 AUD **Framework**: Social Ecological Model of Health	**Focus area**: Health promotion (physical activity) **Eligible projects**: Establish a women’s walking group, support an existing women’s walking group, improve neighbourhood, group or workplace social environment to encourage women’s walking **Eligible recipients**: Community organizations, neighbourhood groups, with priority for women’s groups, such as women with young children, women with careers, culturally and linguistically diverse women, Indigenous women	**Dissemination**: Shared with women’s health networks, local and state community organizations, local and national health departments **Application**: A review committee evaluated applications. Committee members included representatives from the Office for Women, health promoters, health department members **Reporting**: Final report required, report components not described	**Technical Assistance**: A telephone support line was available to applicants and awardees **Website**: Provided details about program, “what’s new” page, application instructions, discussion board for applicants and awardees, project profiles **Partnerships**: Program facilitated partnerships with national stakeholders and a similar national health promotion program for physical activity (10,000 Steps)	Over 100 proposals received, and 48 projects funded in 2-year period **Facilitators**: • Collaboration with 10,000 Steps Program allowed sharing of contacts, cross-promotion, guidance from experienced program staff • Program-specific website facilitated applications, connection amongst awardees and between awardees and program organizers • Public agencies and organizations provided access to experts in women’s and multicultural health **Barriers**: • Payment processing delays Program facilitated contact with priority community groups, e.g., new English speakers	Not described	**Study design**: Descriptive **Quality rating**: Not appraised
Colchamiro, 2015 [52]	**Program**: The Breastfeeding Continuity-of-Care Team (BCCT) catalyst grant program **Organization**: The Massachusetts Department of Public Health **Location**: Massachusetts, USA **Grant size**: Not reported **Framework**: Social Ecological Model of Health	**Focus area**: Maternal and child health (breastfeeding) **Eligible projects**: Projects that support breastfeeding **Eligible recipients**: Municipalities with a higher percentage of low-income, underserved populations	**Dissemination**: Mailing lists to birthing hospitals, Special Supplemental Nutrition Program for Women, Infants and Children (WIC) clinics, partner organizations **Application**: Description of their community and existing capabilities, partnerships with at least 2 community-based organizations, budget, evaluation plan, SWOT (Strengths, Weaknesses, Opportunities, Threats) analysis. Applications were reviewed by program team **Reporting**: Success indicators tracked monthly, including number of eligible births, number of mothers who received support	**Technical Assistance**: Provided by University faculty and community-based health professionals. Monthly meetings to help awardees review progress, troubleshoot challenges **Site Visits**: Members of the program team visited each site at least once **Conferencing**: Meetings to convene all awardees to share successes, best practices	8 proposals received, and 6 projects funded in 10-month period **Facilitators**: • Technical assistance monthly calls and site visits were highly valuable • Conferencing opportunities with awardees fostered camaraderie and sharing of experiences • Media attention provided publicity through a grand opening, government representatives) **Barriers**: • Short timelines challenged project recruitment, organizational approval to apply Program staff learned about communities’ unique strengths and barriers	Collaborative relationships that were formed among the community providers outlasted the grant implementation period. Program staff noted the need to apply for additional funding to maintain services	**Study design**: Descriptive **Quality rating**: Not appraised
Coombe, 2023 [53]	**Program**: Small Planning Grant program and the Community-Academic Research Partnerships Grant Program **Organization**: Detroit Community-Academic Urban Research Center **Location**: Detroit, Michigan, USA **Grant size**: $2000–5000 USD, average $4200 USD **Framework**: Community Based Participatory Research Approach	**Focus area**: Health, public health and social issues (general) **Eligible projects**: Projects that support alleviation of poverty, through building equitable partner relationships, exploring collaborative research interests, conducting community assessments, and disseminating and translating research findings **Eligible recipients**: Community partners, in partnerships with academic researchers	**Dissemination**: Shared with community and research mailing lists, University and Community-Academic Research Network and community organization networks **Application**: Description of project goals, methods, relevance to poverty alleviation, partners, timeline, budget and letters of support. Applications were rated by committee of academic and community partners. Committee had opportunity to request additional information or suggest modifications prior to final decision **Reporting**: Mid-year report provided opportunity to share needs for assistance, and a final report	**Technical Assistance**: Provided on request by program staff **Training**: Workshops providing introduction to community based participatory research, program overview, partnership development and evaluation, and dissemination **Conferencing**: Introductory meetings to convene all awardees. Final meeting to share findings and next steps for sustaining efforts	50 projects funded **Facilitators**: • Conferencing time valuable for partnership development, learning from experts, shared learning with other project teams • Ongoing technical assistance was helpful Keys to building inclusive, equitable partnerships include providing time and capacity building support to build relationships and power-sharing processes	At 1–3 years following program, nearly half of projects had secured additional funding and were planning additional projects. More than half had established a steering committee or partnership infrastructure	**Study design**: Qualitative **Quality rating**: Moderate
Crespo, 2011 [54]	**Program**: Appalachian Coalition **Organization**: Appalachian Regional Commission **Location**: Appalachian counties, USA **Grant size**: $10 000 USD **Framework**: Rural Appalachian Model, adapted from Model for coalition development	**Focus area**: Diabetes prevention and management **Eligible projects**: Promoting healthy eating, physical activity, chronic disease self-management and awareness building **Eligible recipients**: Members of Appalachian communities	**Dissemination**: Not described **Application**: Description of diabetes issues in community. Applications ranked based on applicant group diversity and understanding of public health approach to diabetes **Reporting**: Quarterly reports of numbers of participants	**Training**: 2-day workshop to develop measurable objectives and action plan **Conferencing**: Awardees gather annually to present on their projects **Site Visits**: Program staff visited project sites	66 projects funded **Facilitators**: • Non-traditional application process where objectives and plan are developed during a workshop increased reach to community partners • Awarding full amount upfront was helpful for awardees	58 projects have been sustained past initial funding	**Study design**: Descriptive **Quality rating**: Not appraised
Dafilou, 2022 [55]	**Program**: Community Catalyst Grants **Organization**: Lindy Family Foundation through The Philadelphia Collaborative for Health Equity (P-CHE) **Location**: Philadelphia, Pennsylvania, USA **Grant size**: $50 000 USD **Framework**: World Health Organization Social Determinants of Health Framework	**Focus area**: Mental health and trauma, safety, housing, built environment **Eligible projects**: Engage community with at least one of mental health; trauma, safety, and violence, e.g., developing a community-centred trauma training curriculum; housing, e.g., forming a housing trust; and built environment, e.g., building a park **Eligible recipients**: Latino community of Philadelphia	**Dissemination**: Call for applications announced at community photovoice exhibition **Application**: Application requirements not described. Panel of unaffiliated grant reviewed ranked applications, prioritizing those which addressed findings at photovoice exhibition **Reporting**: Program evaluation not described	**Technical assistance**: Provided but not described **Training**: Policy and advocacy workshop conducted online over 2 weeks	12 projects were funded Allowing community to determine focus of grant funding lead to community ownership of projects. Planning several steps ahead allowed for community involvement in decision-making at each step	Program staff worked with awardees to secure additional funding to sustain projects	**Study design**: Descriptive **Quality rating**: Not appraised
Goodman, 2018 [56]	**Program**: Step Up to Leadership **Organization**: Missouri Association of Community Action and University of Missouri **Location**: Missouri and Illinois, USA **Grant size**: Max. $500 USD **Framework**: Social Cognitive Theory	**Focus area**: Health and social issues (general) **Eligible projects**: Address community issues, e.g., health fairs, farmers markets, community gardens, car seats for low-income mothers **Eligible recipients**: non-profit organizations, business managers, local government officials, church leaders	**Dissemination**: Not described **Application**: Brief description of project and need, expected community impact, budget, list of community partners. Applications reviewed by program staff and board members **Reporting**: Summary of accomplishments, benefits to community, lessons learned, and plans to continue project	**Training**: 12-week leader development program for understanding and embracing diversity, serving on boards of directors, participating in community meetings, and applying for minigrants	18 proposals received, 16 were funded Participants reported increased skills, e.g., leadership, grant writing, increased self-efficacy, and enhanced community involvement Support for applicants throughout grant process was critical in developing skills required to plan and lead projects	Participants noted their acquired grant writing skills were transferable to applying for additional grants	**Study design**: Qualitative **Quality rating**: Moderate
Grossman, 2019 [57]	**Program**: No formal name **Organization**: State health departments, funded by Centers for Disease Control and Prevention **Location**: California, Florida, Illinois, New Hampshire, Oregon and Wisconsin, USA **Grant size**: $7700- 28 500 USD annually **Framework**: Centers for Disease Control and Prevention’s (CDC’s) Building Resilience Against Climate Effects (BRACE) framework	**Focus area**: Environmental health (climate change preparedness) **Eligible projects**: Improving community resilience to climate change, extreme weather; response to health consequences of climate change **Eligible recipients**: Local health departments	**Dissemination**: Request for proposals shared with local health departments **Application**: Requirements not described. Selection based on capability to implement proposed projects **Reporting**: Quarterly and final reports of successes, challenges and recommendations for future programs	**Technical Assistance**: Guidance for accessing and summarizing data on health, social vulnerability and health **Training**: Webinars and in-person workshops were provided	18 projects were funded Awardees reported that training increased knowledge and skill for partnership development, planning and vulnerability assessment **Barriers**: • Awardees found planning difficult due to uncertainty of continued funding	Awardees noted the 1-to 2-year grant duration was insufficient to demonstrate impact that would help secure additional funding	**Study design**: Cross-sectional **Quality rating**: Moderate
Hickey, 2015 [58]	**Program**: Literacivic **Organization**: Youngballymun **Location**: Ballymun, Northern Dublin, Ireland **Grant size**: €200–4000 EUR, depending on project type **Framework**: None	**Focus area**: Youth wellbeing and learning **Eligible projects**: Capacity building for leadership, communications, advocacy; community celebrations or events **Eligible recipients**: Neighbourhood groups, services and organizations	**Dissemination**: Posters and brochures distributed locally **Application**: Written proposal, reviewed by an independent committee **Reporting**: Not described	Not described	42 proposals received; 24 projects were funded Awardees reported that funding developed personal skills, community involvement and helped increase access to available services **Barriers**: • Funding likely inaccessible to some potential applicants • Lack of guidance for application	Not described	**Study design**: Qualitative **Quality rating**: Moderate
Honeycutt, 2012 [59]	**Program**: Nutrition Programs that Work **Organization**: The Emory Cancer Prevention and Control Research Network (CPCRN) **Location**: Georgia, USA **Grant size**: $4000 USD **Framework**: RE-AIM (Reach, Efficacy, Adoption, Implementation, Maintenance)	**Focus area**: Health promotion (nutrition) **Eligible projects**: 1 of 2 programs, Body & Soul for churches and Treatwell 5-a-Day for workplaces **Eligible recipients**: Churches and workplaces	**Dissemination**: Distributed to eligible organizations locally **Application**: Requirements not described. Committee of Community Advisory Board members rated applications according to fidelity to the program, organizational capacity for implementation, and diversity of the organization **Reporting**: Not described	**Technical Assistance**: Bi-monthly teleconferences between program staff and awardees. Email and telephone support provided as requested **Networking Facilitation**: Partnerships with Community Advisory Board members	17 proposals received; 7 projects were funded **Facilitators**: • Technical assistance was necessary and found helpful by awardees • Aligning projects to eligible organizations’ mission statements	All awardees reported intent to continue at least some activities. Several were interested in expanding Sustainability was associated with adaptability of projects, having project champions, alignment with organization’s mission, perceived benefits and stakeholder support	**Study design**: Qualitative **Quality rating**: High
Kegler, 2015 [60]	**Program**: Cancer Prevention and Control Research Networks (CPCRN) Mini-Grants Program **Organization**: Centers for Disease Control and Prevention and National Cancer Institute **Location**: Georgia, South Carolina and Texas, USA **Grant size**: $1000–10 000 USD, average $6250 USD **Framework**: Interactive Systems Framework	**Focus area**: Cancer prevention **Eligible projects**: Adaptations of evidence-based interventions for cancer prevention listed on Research-Tested Intervention Programs database or from research literature **Eligible recipients**: Community-based organizations, faith-based organizations, schools, worksites	**Dissemination**: Not described **Application**: Included organizational capacity to implement project, including leadership and experience. Proposals assessed according to fidelity of work plan to original evidence-based intervention, plans for adaptations, community needs and potential impact, budget justifications and evaluation plan **Reporting**: Final reports required but not described	**Technical Assistance**: Research fellows supported application development. Fellows convened with awardees monthly for guidance with administrative of budget challenges and implementing and adapting interventions **Training**: Workshops provided to potential applicants on finding, selecting, adapting evidence-based interventions. Workshops provided to awardees on implementing and sustaining projects	105 proposals received; 44 projects were funded 2007–2014 Most proposals were based on selected interventions featured on the Research-Tested Intervention Programs database, rather than from other research literature None of the awardees conducted evaluations as described by selected interventions. This limited evaluation of effectiveness, especially when interventions were adapted to different contexts or populations	Awardees were most successful in sustaining projects when they were able to establish new partnerships. In several cases, partners continued projects after the grant period	**Study design**: Descriptive **Quality rating**: Not appraised
Main, 2012 [61]	**Program**: Community Engagement Pilot Grants Program **Organization**: University of Colorado Denver **Location**: Colorado, USA **Grant size**: $10 000 or $30 000 USD, depending on project type **Framework**: None	**Focus area**: Health (general) **Eligible projects**: Address priority health issues, e.g., childhood chronic conditions, social and emotional health, or cardiovascular disease prevention **Eligible recipients**: Community representatives, academic researchers	**Dissemination**: Through university partners and community partners identified by The Partnership of Academicians and Communities for Translation Council **Application**: Key sections included project focus, outcomes, partnerships, community engagement plan and budget. Dyad of community and academic representatives scored applications. Nonfunded applications were provided feedback and encouraged to reapply **Reporting**: 6-month and final report describing partnerships, community engagement, results, lessons learned and future plants. Awardees also regularly reported on their budget	**Technical Assistance**: Webinar for potential applicants on proposal requirements **Training**: Awardees attended 8-h workshop on community engagement	36 projects were funded Initially, projects could address any health topic. Projects eligibility was revised to priority topics to maximize potential impact Following challenges during the first funding cycle, the application period was extended and additional technical assistance was provided to applicants to facilitate the application process	The initial investment of $272 742 led to over $2.8mil in new funding to several awardees	**Study design**: Descriptive **Quality rating**: Not appraised
Mayberry, 2009 [62]	**Program**: Pfizer Foundation Southern HIV/AIDS Prevention Initiative **Organization**: Pfizer Foundation contracted with Morehouse School of Medicine Prevention Research Center **Location**: Southern USA **Grant size**: Not reported **Framework**: Empowerment Evaluation Framework	**Focus area**: HIV prevention **Eligible projects**: HIV education and prevention programs **Eligible recipients**: Community-based organizations in multicultural, urban and rural communities	**Dissemination**: Not described **Application**: Not described **Reporting**: Not described	**Technical assistance**: Phone calls and site visits from program staff helped guide awardees **Training**: Initial focus for training was on developing logic models and measurable objectives. Subsequent workshops focused on skills for planning, implementing and evaluating projects. Feedback was gathered from awardees to inform focus of workshop sessions	69 projects were funded **Facilitators**: • Initial needs assessment and ongoing solicitation of feedback from awardees ensured technical assistance met each team’s needs • Regular communication allowed for targeted learning opportunities Regular interactions allowed integration of evaluation into activities	Increased capacity of awardees to implement and evaluate projects contributed to project sustainability	**Study design**: Single group pre-post **Quality rating**: High
McCracken, 2013 [63]	**Program**: Community Health Intervention Program (CHIP) mini-grants initiative **Organization**: South Carolina Cancer Prevention and Control Research Network (SC-CPCRN) **Location**: South Carolina, USA **Grant size**: $10 000 USD **Framework**: None	**Focus area**: Cancer prevention **Eligible projects**: Adaptations of evidence-based interventions for cancer prevention listed on Research-Tested Intervention Programs database **Eligible recipients**: Community-based organizations	**Dissemination**: Not described **Application**: Requirements not described. Panel of faculty, staff and community partners rated applications according to how well the proposal, evaluation and timeline aligned with the original evidence-based intervention. Applicant interest and experience, support from leadership, community need and diversity were considered **Reporting**: Regular updates and reports to program liaisons. A mini-grant report template was developed to capture quantitative and qualitative information. Awardees presented findings at a program event	**Technical assistance**: In-person and virtual sessions for potential applicants. Program staff provided ongoing guidance and oversight	12 proposals received; 3 projects were funded **Facilitators**: • Collaboration, communication and trust between program staff and awardees • Community engagement **Barriers**: • Competing priorities for community needs vs. research and evaluation processes	Not described	**Study design**: Descriptive **Quality rating**: Not appraised
Nieves, 2020 [64]	**Program**: Health in Action Project **Organization**: New York State Health Foundation and Mount Sinai Health System **Location**: East Harlem, New York, USA **Grant size**: $25 000 USD **Framework**: Health Department’s framework for community engagement	**Focus area**: Health, public health and social issues (general) **Eligible projects**: Designed to improve community health **Eligible recipients**: Non-profit and community organizations	**Dissemination**: Request for proposals shared with local non-profit and community organizations **Application**: Requirements not described. Panel of community members assessed proposals. Panel members required to describe interest in participation and thoughts on local health issues. Panel chose short list of proposals, which were presents to the public. Successful applicants selected by vote **Reporting**: Mid-year and final reports of project metrics, successes, challenges, lessons learned, partnerships	**Training**: Workshops on community advocacy, civic engagement. Quarterly capacity building activities **Conferencing**: Awardees convened quarterly to network, share successes and challenges	20 proposals were received, 16 were selected for short list, 11 projects were funded **Barriers**: • Challenging to implement a process that was new for both program staff and community members • Time allotted for proposals and award selection, training, was insufficient • Health impact of funded projects was not evaluated Establishing new and strengthening existing partnerships facilitated connection to communities. Funding to support organizational capacity building expanded awardees’ reach within communities	Partnerships between awardees and other organizations expected to help sustain projects	**Study design**: Qualitative **Quality rating**: High
Paberzs, 2014 [65]	**Program**: Community–University Research Partnership (CURES) Award **Organization**: Michigan Institute for Clinical and Health Research (MICHR) Community Engagement Program **Location**: Michigan, USA **Grant size**: Max. $25 000 USD **Framework**: None	**Focus area**: Health (general) **Eligible projects**: Projects designed to improve health outcomes in at-risk populations **Eligible recipients**: Dyads of an academic teams and a community based organization	**Dissemination**: Not described **Application**: Research plan outlining objectives, study design, methods and potential significance, as well as description of partnership, dissemination plan and community need. Applications scored by Scientific Review Committee for significance, investigators, innovation, approach, environment and overall impact, and by Community Engagement Coordinating Council using 9-point National Institutes of Health scoring scale. Scores were averaged in final decision. Nonfunded applications were provided feedback and encouraged to reapply **Reporting**: Not described	**Technical Assistance**: Potential applications could receive consultations to support application development. Program staff available to awardees to guide partnership development and adherence to ethics board requirements,	50 proposals received; 16 projects were funded Application review procedures were adjusted over time. Changes included assigning community members, in addition to faculty members, as lead reviewers. A formal process to report and manage conflicts of interest was established. Definitions of terms and criteria were clarified. Most reviewers agreed that piloting the review process would have been beneficial	A description of project sustainability was required for the application and scored by reviewers	**Study design**: Descriptive **Quality rating**: Not appraised
Pearson, 2020 [66]	**Program**: Shaheed DuBois Community Grant Program **Organization**: HERCULES Exposome Research Center **Location**: Atlanta, Georgia, USA **Grant size**: $2500 USD **Framework**: None	**Focus area**: Environmental health **Eligible projects**: Any environmental health-focused project, e.g., pollution, social stressors, built environment, healthy food access, water pollution, and waste disposal or illegal dumping **Eligible recipients**: Smaller, neighbourhood-level grassroots organizations	**Dissemination**: Not described **Application**: Statement of community need, description of project and how it meets community need, project timeline, budget, leadership support and resources available. Scored according to a rubric by one community and one academic representative **Reporting**: Quarterly, then revised to biannual standard report forms documenting activities, outcomes, successes, challenges and needed support. Awardees present accomplishments and next steps at annual program event	**Technical assistance**: Support provided during application process and project implementation, both through regularly scheduled calls and site visits and as requested. A sample invoice was provided to guide awardees through invoicing **Networking facilitation**: Program staff connected awardees to available partners and experts **Training**: Workshops for program implementations, evaluation, budgets and invoicing	13 projects were funded Awardees valued technical assistance provided. Some awardees noted they were unaware of types of support technical assistance could provide Awardees valued opportunities to meet other awardees	All awardees planned to continue or expand their projects. Several had secured additional funding and established partnerships to support sustaining projects	**Study design**: Qualitative **Quality rating**: Moderate
Ramanathan, 2018 [44] Tamminen, 2014 [67]	**Program**: Teen Challenge Program **Organization**: ParticipACTION, supported by Coca-Cola **Location**: Canada **Grant size**: Max. $500 CAD **Framework**: None	**Focus area**: Health promotion (physical activity) **Eligible projects**: Physical activity programs for adolescents, e.g., costs associated with facilities, equipment, instruction, uniforms, prizes or promotional materials **Eligible recipients**: Community organizations	**Dissemination**: Online ads; shared with provincial and territorial program coordinators, and schools **Application**: Demonstrate capacity to promote or support physical activity for adolescents. Reviewed by provincial and territorial program coordinators **Reporting**: Annual survey of provincial and territorial program coordinators, annual survey and database of awardees	**Website**: Provided tools and resources, e.g., physical activity statistics, guidance for engaging adolescents, infographics and promotional posters for download	Approximately 75% of proposals were funding. In total, 3128 projects were funded **Facilitators**: • Flexibility of funding allocation • Funded status increased perceived credibility and facilitated partnerships **Barriers**: • Applicants found the online registration process difficult	For many funded projects, the purchase of equipment will allow projects to continue	**Study design**: Qualitative **Quality rating**: Moderate
Schmidt, 2009 [68]	**Program**: No formal name **Organization**: The Hague Municipal Health Services **Location**: The Hague, Netherlands **Grant size**: €500–3500 EUR **Framework**: None	**Focus area**: Health promotion (physical activity, nutrition) **Eligible projects**: Innovative projects related to physical activity or nutrition **Eligible recipients**: Community organizations, resident groups	**Dissemination**: Not described. Most awardees were members of the program panel **Application**: Requirements not described. Reviewed by neighbourhood panels consisting of health services staff and community workers, e.g., librarians, dietitians, community centre staff, youth health care nurses, etc **Reporting**: Standardized report describing the project, its progress and outcomes	**Conferencing**: Most awardees were members of program panels that met regularly	61 projects were funded **Facilitators**: • Neighbourhood panels facilitated access to “hard-to-reach” community members • Experienced moderators chaired panel discussions **Barriers**: • Application review guidelines were vague and review panels applied criteria inconsistently, e.g., sustainability ratings were based on neighbourhood empowerment for some applications and financial stability for others Public participation in projects was limited	At least 26 projects were sustained, most through participation fees	**Study design**: Qualitative **Quality rating**: Moderate
Sharpe, 2015 [69]	**Program**: Community Advocacy and Leadership Program **Organization**: Prevention Research Center **Location**: South Carolina, USA **Grant size**: $5000 USD **Framework**: None	**Focus area**: Built environment **Eligible projects**: Changes to build environment to support physical activity, e.g., building walking track or playground **Eligible recipients**: Community organizations in priority areas	**Dissemination**: Call for proposals shared with community organizations in priority areas **Application**: Letters of intent approved prior to full application. Application included project description, team experience and plans to involve the community. Additional $1250 in funding required. Program leadership reviewed and ranked applications, interviewed applicants **Reporting**: Documentation of spending and final report that included photos	**Technical assistance**: Program staff met with awardees monthly to problem solve, identify resources or referrals **Training**: 8 workshops for applicants and awardees. Topics included grant writing, leadership, advocacy sustainability, strategic planning **Networking facilitation**: Awardees were connected with community organizations	2 projects were funded Workshops provided networking opportunities for applicants and awardees Applicants and awardees had limited writing and computer skills **Facilitators**: • Accommodated limitations in discreet manner	Not described	**Study design**: Mixed methods **Quality rating**: Low
Smallwood, 2015 [70]	**Program**: Community Empowerment Center Funded Mini Grant Project **Organization**: Community Empowerment Center **Location**: Columbia, South Carolina, USA **Grant size**: Max. $12 000 USD **Framework**: None	**Focus area**: Social issues **Eligible projects**: Any projects that address community social issues **Eligible recipients**: Local public health units, residents	**Dissemination**: Not described **Application**: Letters of intent approved prior to full application. Application included plans to sustain project beyond funded period. Graduate students reviewed applications and convened a panel to select successful applications **Reporting**: Weekly progress updates, monthly reflection on successes and barriers, monthly financial report, and final report	**Technical assistance**: Two sessions for applicants to receive help developing application **Training**: Workshops on implementation of community change interventions. Additional “power up” skill-building sessions on specific topics **Conferencing**: Program staff met monthly with awardees to discuss strategies for community engagement **Website**: Mentioned as tool to establish community presence, but not described further	10 letters of intent received, 6 full proposals received, 3 projects were funded It was valuable for awardees to meet monthly and learn from others’ successes and challenges. Awardees with later start dates benefitted from learning from awardees who were further along with projects Additional training for project management and evaluation needed	1 project continued past the funding period, although at a reduced capacity. Awardees reported difficulty sustaining project when funding ended	**Study design**: Descriptive **Quality rating**: Not appraised
Soares, 2014 [71]	**Program**: Community Access to Child Health (CATCH) Program **Organization**: American Association of Pediatrics Division of Community-based Initiatives **Location**: USA **Grant size**: Average $10 213 USD **Framework**: None	**Focus area**: Health (general) **Eligible projects**: Planning or implementation of projects to improve child health at community level **Eligible recipients**: Pediatricians	**Dissemination**: Not described **Application**: Description of community and proposed intervention. Applications scored by 3 program staff **Reporting**: Routine progress updates and follow-up to assess sustainment at 2-years post-award	**Technical Assistance**: Guidance provided on to conducting a needs assessment, community asset mapping, developing resources, community coalition building, and project evaluation **Website**: Web-based application facilitated application process and ongoing data collection. A public-facing site provides information about the granting program and previous projects	731 proposals received; 201 projects were funded 87% of awardees obtained technical assistance. Most (63% received grant writing support or obtained information/materials (44%)	Many partnerships were sustained 2 years after funding period, and many new partnerships had been formed	**Study design**: Qualitative **Quality rating**: Moderate
Tendulkar, 2011 [72]	**Program**: Harvard Catalyst Community Based Participatory Research Partnership Program **Organization**: Harvard Clinical and Translational Science Awards **Location**: Massachusetts, USA **Grant size**: Max. $50 000 USD **Framework**: None	**Focus area**: Public health and health (general) **Eligible projects**: Any projects related to health, such as nutrition, cancer screening, youth sex education, air quality, etc **Eligible recipients**: Community organizations	**Dissemination**: Request for proposals shared with networks of community partners **Application**: Written proposal required. Reviewed by researcher and community partner **Reporting**: Not described	**Technical Assistance**: Information session provided to applicants to review proposals and provide feedback **Training**: Workshops on negotiating equitable community-research partnerships, research ethics	10 proposals received; 4 projects were funded Lessons learned included allowing sufficient time to develop partnerships and proposals, and to solicit and respond to feedback from awardees	Not described	**Study design**: Descriptive **Quality rating**: Not appraised
Thompson, 2010 [73]	**Program**: No formal name **Organization**: Hispanic Community Network to Reduce Health Disparities **Location**: Lower Yakima Valley, Washington, USA **Grant size**: $2500–3500 USD **Framework**: None	**Focus area**: Cancer prevention **Eligible projects**: Any projects related to cancer prevention **Eligible recipients**: Community groups or organizations	**Dissemination**: Request for proposals shared with community organizations **Application**: Statement of work, contribution of project to program goals, applicant qualifications, evaluation plan, and budget. Panel of community advisory board scored applications according to scientific merit, applicant capability, project contributions, adequacy of evaluation, and suitability of budget **Reporting**: Not described	**Technical Assistance**: 4-h session to assist with application process	12 proposals received; 10 projects were funded The application process was challenging for most applicants due to language and education barriers	Sustainability was a challenge for many projects	**Study design**: Qualitative **Quality rating**: Moderate
Tompkins, 2022 [74]	**Program**: No formal name **Organization**: West Virginia state health department **Location**: West Virginia, USA **Grant size**: $196 369 USD was dispersed to 65 organization **Framework**: Social Ecological Model and the Health Impact Pyramid	**Focus area**: Health promotion (physical activity, nutrition) **Eligible projects**: Interventions that address policy, systems, and environmental changes **Eligible recipients**: Non-profit and private organizations, schools, local health departments	**Dissemination**: Not described **Application**: Description of change strategies, how they will address inequities, partnership with Health Connection organization, planning for sustainability. Application review process not described **Reporting**: Not described	**Technical Assistance**: Assistance and resources provided but not described **Website**: Contained request for proposals and resources for applicants and awardees	65 projects were funded Evaluation of project outcomes was challenging due to heterogeneity of settings, activities, timelines and project foci Structural capacity of organizations varied, many awardees were not trained in public health or related fields Early and ongoing communication with awardees was valuable	Sustainability addressed by most awardees. Many applied for additional funding. Some integrated project activities into existing practices	**Study design**: Descriptive **Quality rating**: Not appraised
Vanderpool, 2011 [75]	**Program**: Appalachia Community Cancer Network (ACCN) grant program **Organization**: National Cancer Institute (NCI) **Location**: Appalachian region, USA **Grant size**: $3500 USD **Framework**: None	**Focus area**: Cancer education **Eligible projects**: Evidence-based cancer prevention intervention **Eligible recipients**: Community organizations, local coalitions, faith-based organizations, social service agencies, health clinics	**Dissemination**: Not described **Application**: Narrative statement of need, work plan, evaluation plan, budget with justification. Formal review of applications not described **Reporting**: Final report required	**Technical Assistance**: Support for proposal development and program implementation **Training**: Workshops based on NCI’s curriculum, Using What Works: Adapting Evidence-Based Programs to Fit Your Needs, to help awardees identify, adapt and implement evidence-based interventions **Website**: Web portal provided links to sources of research-tested interventions, guidance on program development	13 proposals received; all 13 projects were funded Most applications used Cancer Control P.L.A.N.E.T. website to identify evidence-based interventions Awardees found technical assistance and training helpful Some awardees felt that evidence-based interventions did not fit their local needs or found the process overwhelming Interventions adapted by adjusting timelines, tailoring materials, planning additional activities, combining multiple programs, and modifying evaluation plans	Projects were not sustained in their entirety, but 4 awardees continued to use materials for other health-related activities	**Study design**: Qualitative **Quality rating**: High
Vines, 2011 [76]	**Program**: Carolina Community Network (CCN) **Organization**: Community Network Program (CNP) **Location**: North Carolina, USA **Grant size**: Max. $10 000 USD **Framework**: Community Grants Program (CGP) model	**Focus area**: Cancer prevention **Eligible projects**: Cancer education or evidence-based intervention for cancer prevention **Eligible recipients**: Community organizations, faith-based organizations, health care agencies	**Dissemination**: E-mail distribution lists, information sessions in community **Application**: Description of project and evaluation plan. Pairs of community representatives and researchers scored applications. Score, project type, geographic region and potential impact considered in choosing awardees **Reporting**: 6-month progress report and 12-month final reports required	**Technical Assistance**: Start-up meetings upon awardee selection, to address issues raised by review committee, orient funding processes, and potential collaboration with other awardees **Training**: Session to orient applicants to the Community Grants Program model and application review process **Conferencing**: Monthly calls between awardees and program staff **Networking facilitation**: Program staff connected awardees with similar projects	36 proposals received; 15 projects were funded Lessons learned: • Power imbalance between academic researchers and community organizations managed by giving organizations ability to choose projects and strategies, more information on academic finances Approaches to partnerships must be tailored to diverse needs to community organizations	3 projects were funded again through re-application for a grant	**Study design**: Descriptive **Quality rating**: Not appraised
Washington, 2022 [77]	**Program**: No formal name **Organization**: National Center on Health Physical Activity and Disability **Location**: Birmingham, Alabama, USA **Grant size**: Max. $20 000 USD **Framework**: None	**Focus area**: Health promotion (general) **Eligible projects**: Inclusive neighbourhood programs for people with disabilities and broader community **Eligible recipients**: Neighbourhood groups	**Dissemination**: Promoted through organization’s website and social media, asked partners to promote to their networks **Application**: Description of planned program, plans to include people with disabilities, partnerships supporting implementation. Scored by graduate students according to statement of need, program description, experience, partnerships, organizational capacity, evaluation plan. Scores were averages across reviewers **Reporting**: Not described	**Technical assistance**: Interested communities were provided with virtual sessions to discuss granting program **Training**: Mandatory 1.5-h community engagement workshop focused on innovative community engagement strategies, community strategies, engaging people with disabilities. Training was recorded and made available to awardees **Website**: Information about the program posted on the funding organization’s website	5 projects were selected but 2 awardees declined their awards due to funding requirements. 3 projects received funding Awardees shared expertise and experiences in working with people with disabilities	Partnerships were seen as the sustainable component of the program	**Study design**: Descriptive **Quality rating**: Not appraised
Wingfield, 2012 [78]	**Program**: SUCCEED Legacy Grant Program **Organization**: Racial and Ethnic Approaches to Community Health (REACH) **Location**: Georgia, North Carolina and South Carolina, USA **Grant size**: $20 000 USD **Framework**: None	**Focus area**: Cancer prevention **Eligible projects**: Evidence-based breast and cervical cancer interventions with focus on reducing health inequities for Black women **Eligible recipients**: Community organizations, faith-based organizations	**Dissemination**: Not described **Application**: Written proposals scored by review committee according to overview of community needs, organizational capacity, program description, partnerships, evaluation plan, budget and justification. Nonfunded applications were provided feedback and encouraged to reapply **Reporting**: Semi-annual and year-end reports on progress toward objectives, technical assistance received, recommendations for the granting program	**Technical Assistance**: Annual webinars share information about the grant program and application process. Ongoing support provided to awardees for evaluation planning, implementing work plans, and developing reports **Training**: Workshops provided but not described **Networking facilitation**: Program staff connected awardees with relevant community organizations	9 projects were funded Awardees found that program staff provided critical support in identifying resources and opportunities On-going training with awardees was required as projects progressed Face-to-face interactions between awardees and program staff facilitated trust Proposed timelines were difficult for many awardees to follow	Awardees were supported in applying for additional funding to sustain projects	**Study design**: Descriptive **Quality rating**: Not appraised
Wyatt, 2011 [79]	**Program**: Somos Fuertes: Strong Women Making Healthy Choices **Organization**: Not described **Location**: Southwestern USA **Grant size**: $600 USD **Framework**: Social Learning Theory, Role Theory, and Diffusion of Innovations	**Focus area**: HIV prevention **Eligible projects**: HIV education events **Eligible recipients**: Registered university student organizations	**Dissemination**: Applications distributed to student organization mailboxes and e-mail addresses. Ad posted in student newsletter **Application**: Proposed activities, signed agreements to fulfill grant requirements, answers to questions about HIV knowledge and education on campus. Applications reviewed by program directors **Reporting**: Results of survey of project participants’ pre- and post-activity HIV knowledge	**Training**: Train-the-trainer workshop on effective HIV education, HIV characteristics **Materials**: Evidence-based fact sheets and hand-outs on HIV statistics, condom effectiveness and usage	5 proposals were selected, 4 completed requirements to receive full funding amount Some positive increases in participants’ HIV knowledge and planned safe behaviours	Not described	**Study design**: Single group pre-post **Quality rating**: Low

Approximately one-third of included studies were qualitative in design, (*n* = 13, 37%), and explored program implementation from the perspectives of program staff and/or awardees through interviews or open-ended survey questions [44, 45, 53, 56, 58, 59, 64, 66, 68, 69, 71, 73, 75]. Qualitative analyses of responses identified facilitators, barriers and lessons learned in program implementation. One article used a mixed methods design [69]; however, only the quantitative portion of the study was not relevant to this review and therefore the study was analysed and appraised as a qualitative study. Of the qualitative studies, four were rated as high quality [45, 59, 64, 75], eight as moderate quality [44, 53, 56, 58, 66, 68, 71, 73], and one as low quality [69]. Quality assessments are included in Supplemental Table A2c.

There were also three studies that used a quantitative design. Two were two single-group pre-post evaluations [62, 79] of which one was rated high quality [62] and the other was rated low quality [79], as shown in Supplemental Table A2a. The other study was cross-sectional and rated low quality [57], see Supplemental Table A2b for the detailed quality assessment.

### Program Characteristics

Most of the 35 programs were based in the USA (*n* = 31, 89%), while the remaining programs were based in Canada [44], Australia [51], Ireland [58], and the Netherlands [68]. In terms of scope, two programs were available to community groups nationally, [44, 71], while two-thirds of programs, *n* = 23 (66%) were offered across one or several states [45, 46, 48, 49, 51, 52, 54–57, 59–63, 65, 69, 72, 74–76, 78, 79], and ten (29%) were available within local communities [47, 50, 53, 58, 64, 66, 68, 70, 73, 77].

In describing program development, approximately half (*n* = 19, 54%) of community granting programs cited one or more models or frameworks. There was little consistency, with 15 different frameworks cited across 19 programs. Three programs developed original frameworks or adapted frameworks to their contexts [48, 54, 64]. The Socioecological Model [80] was cited by four programs [50–52, 74] and the Community-Based Participatory Research model [81] was cited twice [47, 53]. Of the 19 community granting programs that cited a framework or model, 12 reported positive health, community or social outcomes and 11 reported on outcomes related to sustainability, such as securing additional funds, strengthened applications for additional funds, and partnerships (Table 1).

Ten (29%) programs were developed in partnership with academic or research institutions, offering grant funding for community participatory research projects [46, 47, 53, 60, 61, 64, 65, 72, 73, 76]. Community projects funded by these programs were similar to other community granting programs but required ethics review and additional evaluation for research outcomes.

Programs reported grant size differently, where some reported total funding pool amount, the amount available for individual grants, or both. The smallest total funding pool was reported as $10 000 USD (approximately $13 000 CAD). This review excluded community granting programs with funding pools over $500 000 CAD. Individual grant size varied considerably, with awards as small as €200 (approximately $300 CAD) and as large as $25 000 USD (approximately $34 000 CAD).

### Project eligibility

#### Program Focus

Community granting programs were designed to address broad or narrow scopes of community health priorities. Approximately two-thirds of programs (*n* = 24, 69%) focused on a specific public health topic area, including physical activity and nutrition [44, 45, 51, 59, 68, 74, 77], cancer prevention [49, 60, 63, 73, 75, 76, 78], environmental health [50, 57, 66, 70], HIV prevention [62, 79], breastfeeding [52], diabetes education and prevention [54], mental health, trauma, safety and violence [55] and the built environment [69]. The remaining 11 (31%) programs were broader in their focus, and accepted proposals for any aspect of community health.

#### Evidence-based Proposals

Some community granting programs required that project proposals were based on evidence (*n* = 7, 20%). Granting programs implemented this requirement in different ways, defining evidence as either data for community needs or research-based interventions. Two programs required proposals to address priority needs for their communities, based on community-level data [47, 50]. The Community Health Innovation Awards used a community survey to identify priority concerns and accepted project proposals that addressed these concerns [47]. The Environmental Public Health Tracking Network program shared community data through an online portal to inform project proposals [50]. Five programs required that project proposals were based on research evidence and referred applicants to databases of evidence-based interventions [45, 60, 63, 75, 78]. Health promotion community granting programs referred applicants to the Community Preventive Services Task Force’s Community Guide to Preventive Services Creating or Improving Places for Physical Activity, the Centers for Disease Control and Prevention’s Recommended Community Strategies and Measurements to Prevent Obesity in the United States. Cancer prevention community granting programs referred applicants to the National Cancer Institute’s Cancer Control P.L.A.N.E.T. website and Research Tested Intervention Programs database. Of the seven community granting programs that required evidence-based project proposals, four reported positive health or community outcomes.

#### Eligible Community Groups

Community granting programs offered grants to various types of community groups. These include non-profit organizations, neighbourhood associations, community health centres, educational institutions, student organizations, faith-based organizations, state, local, or county public health departments, and other nongovernmental agencies.

### Grant Program Administration

#### Dissemination

Community granting programs used various methods to disseminate information about available grant opportunities. Calls for applications were shared both digitally (through listservs, granting program websites and partner websites) and physically (with paper brochures and posters).

#### Application requirements

Application requirements varied across community granting programs. Common application elements included a statement of purpose, description of the project or project work plan, statement of community need, the potential impact or description of how the project addresses community needs, the team’s experience and capacity to implement the project, list of partners and their roles, anticipated health outcomes, timeline, evaluation plan, and a budget with justification.

#### Application review

Several (*n* = 4, 11%) community granting programs required that applicants submit a letter of intent prior to submission of a full proposal [46, 47, 69, 70]. Less than a third (*n* = 10, 29%) of programs used a formal rubric to rate applications. The 9-point National Institutes of Health scoring scale [65] was used by three programs, of which two programs modified the scale to meet their needs [46, 76]. One program invited applicants for interviews with the selection committee [69], while another program required a presentation by applications to the selection committee [47]. The application review process was not described by the remaining programs.

For most programs, selection committees consisted of program leadership or staff. For community-research partnership programs, both community and research representatives reviewed applications and informed selection. Two programs involved community members in the application review process [55, 64]. To encourage nonfunded applicants to reapply, three programs provided feedback on non-funded applications [61, 65, 78].

#### Reporting requirements

Twenty-seven studies described reporting requirements for funded projects (75%), which typically included mid-project and final budget updates and reports on progress toward project goals. Mid-project updates often provided opportunity for awardees to share concerns and obtain additional support from program staff. Three community granting programs concluded their programs by convening all awardees at an event to present their completed community projects [46, 63, 66].

### Program components

#### Technical Assistance

Most commonly, programs provided technical assistance to applicants or awardees (*n* = 25, 71%). Program staff provided technical assistance to address various needs and challenges, including application development, program planning and implementation, or evaluation. Technical assistance was provided to interested applicants to support application development by 14 (40%) programs. This includes seven programs that held virtual or in-person information sessions [61, 63, 70, 72, 73, 76, 78] and seven programs that made program staff available to provide support on an ad hoc basis [47, 51, 60, 65, 66, 75, 77]. For awardees, technical assistance supported all stages of project planning, implementation, and evaluation. Program staff provided assistance through regularly scheduled meetings [52, 60, 69], on an ad hoc basis [51, 53, 62, 63, 65, 75, 78], or both [48, 59, 66]. Four programs noted that technical assistance was provided to awardees, but do not provide additional details [55, 57, 71, 74]. Program staff for four programs visited project sites to conduct on-site consultations [52, 54, 62, 66]. Finally, one granting program described matching dedicated program staff to funded projects to provide continuous support [50].

#### Workshops and training

Workshops or training was made available to interested applicants or grant awardees by most community granting programs (*n* = 22, 60%). Studies noted that workshops often provided opportunities for program staff and awardees to connect, and for awardees to network and share learning. Workshops focused on topics to support application development and project implementation, including project planning [54, 62, 69], implementation [60, 62, 66, 70], evaluation [48, 53, 54, 62, 66], dissemination [53], partnership development [53, 72], community engagement [61, 77], and budget development [66]. Two programs that required proposals based on research evidence provided workshops on finding, selecting and adapting evidence-based interventions [60, 75], including a workshop based on the National Cancer Institute’s “Using what works” curriculum [75]. Several programs provided workshop sessions focused on social action, including anti-racism and diversity [48, 56], and policy and advocacy [55, 64, 69]. To enhance the long-term sustainability of funded projects, some programs offered sustainability-focused training [60, 69] or workshops to develop grant writing skills in order to support securing additional funding [47, 56, 69]. In response to the diverse needs and strengths of awardees, community granting programs also offered workshops to develop soft skills, such as participating in meetings, serving on boards of directors, leadership, innovative thinking and idea development [47, 56, 69]. Community granting programs that funded community-based participatory research through community-research partnerships also provided workshops on the principles of participatory research and research ethics [53, 72].

#### Websites

Of the 35 programs, only 6 (17%) described a program website to support the community granting program as an online hub to facilitate administration, a collection of digital resources to support applicants and awardees, or both. The website for the Women's Active Living Kits Community Grant Scheme included program details, profiles and updates of funded projects, and a discussion board for applicants and awardees [51]. The Community Access to Child Health Program website was used to collect applications and project reports [71]. The Teen Challenge program website provided awardees with support for community engagement, including guidance on engaging adolescents, infographics, and posters [44]. To support the development of evidence-based proposals, the Appalachia Community Cancer Network program website included links to sources of evidence-based interventions [75]. The Community Empowerment Center Funded Mini Grant Project website was not described in detail [70].

#### Networking facilitation

Program staff were tasked with facilitating connections between grant awardees with similar projects for two community granting programs [50, 76]. Four (11%) programs sought to connect awardees and community partners to leverage existing partnerships within the community [59, 66, 69, 78].

### Outcomes

Outcomes were mostly reported in terms of the granting program, e.g., the number of proposals received and the number of projects funded. However, there were several examples of community impact, health-related outcomes, and outcomes related to sustainability reported.

#### Community outcomes

Overall, positive impacts on the community were reported by community granting programs in qualitative and case report studies. Social cohesion and enhanced community engagement in health-promoting activities were specifically noted [45, 63]. Reports indicated that priority community groups were engaged by community-led projects [51] and that granting programs strengthened their connections with the communities they serve [50, 52, 63, 64, 77].

Most studies did not report on health-related outcomes or specify whether health outcomes were measured in funded projects. For studies that did report on health outcomes, the validity and reliability of measures was not reported. The two studies that reported on health-related outcomes measured environmental health outcomes, and knowledge of health-related topics and of intention to engage in healthy behaviours. This includes the study of the Environmental Public Health Tracking Network granting program, which reported the addition of cooling centres during extreme heat and additional testing of well water during extreme flooding [50]. As well, the study of the Somos Fuertes: Strong Women Making Healthy Choices program reported increased participant knowledge and planned safe behaviours for HIV prevention [79].

Other outcomes reported by studies included beneficial skills for awardees, including project planning and implementation and securing grant funds [46, 56–58]. Programs also reported that awardees developed valuable partnerships to support longer-term goals [46, 49, 52, 53, 60, 64, 66, 71, 77].

#### Sustainability

Project sustainability was typically evaluated at program completion, rather than after a longer term, so most findings reflect the potential sustainability of projects. Only the Community Access to Child Health Program followed up with awardees in the years following project completion and contacted awardees after two years [71].

Several programs (*n* = 6, 17%) noted that awardees were successful in securing additional funding to continue or expand their projects [45, 53, 55, 61, 66, 76]. In addition, awardees with two community granting programs were reported to have submitted applications for additional funding, but it was not noted if these applications had been successful [74, 78]. Awardees from another program noted that the preliminary data gathered during the project was used to strengthen subsequent funding applications [46], although awardees from a different program felt that the short funding period did not provide enough time to collect enough data to support applications [57]. Finally, one community granting program reported that a project was able to use funds to establish a community project that was then funded in the long-term with ongoing participation fees [68].

In addition to reports of additional funding, awardees also reported that through project implementation and participation in workshops provided by the community granting program, they gained valuable and transferable skills for new projects [56, 62]. Partnerships were also noted as a key indicator of project sustainability, reported by eight (23%) of the community granting programs. These partnerships were expected to support projects in the long term and to help generate new community projects [46, 52, 53, 60, 64, 66, 71, 77].

Programs that funded projects that changed the built environment (for example, through the construction or improvement of trails or parks, or projects that purchased equipment for the community) were noted to have inherently longer-term impact as these changes continued to be available after project completion.

### Facilitators and barriers

#### Facilitators

Due to the heterogeneity in reported study outcomes, it was not possible to determine if there were any granting program components with greater contribution to overall program success. Rather, community granting programs reported on facilitators more broadly as they related to various program components and overall implementation. These facilitators were identified by both program staff and grant awardees. For program components, the factors most often cited for project success were the technical assistance and workshops provided by the community granting program [45, 50, 52, 53, 56, 59, 66, 71, 75, 78]. In additional, two programs noted that soliciting ongoing feedback from awardees was critical to informing the technical assistance and workshops offered [62, 72]. Networking amongst awardees often occurred at workshops and was cited as a valuable resource for knowledge sharing [52, 53, 66, 69, 70]. Workshops were also described as an opportunity to build trust between program staff and awardees [78]. For granting programs that hosted a program website, the website was described as a valuable asset that facilitated applications and connections, both amongst awardees and between awardees and program organizers [51].

Engaging the community and responding to community needs were also noted to impact project success. One community granting program emphasized community involvement at all stages of project planning, to ensure projects meet community needs [55]. Another granting program noted that inviting community members to join a program advisory panel helped facilitate engagement with community groups that may have otherwise been difficult to reach [68].

#### Barriers

Program staff and awardees also identified barriers that hindered program administration and project success. Most commonly, timelines were cited as a challenge. Applicants noted that the time between the program’s call for application and its subsequent deadline was not sufficient to complete application requirements [49, 52, 61, 64]. Other awardees noted that the funding period was insufficient to spend the full amount of awarded funds [45, 78].

Application requirements were also reported as a barrier, noting that requirement may not align with the language and education of potential applicants [73]. Awardees from a program that required projects to follow evidence-based interventions noted that interventions available in the research literature did not fit their community’s needs and required significant changes, raising doubts as to their effectiveness [75].

## Discussion

The findings of this review explore many examples of community-driven health or public health projects funded through community granting programs. Findings characterize the scope of projects, grant administration, and outcomes. Evidence for the relative success of programs is less clear, due to the heterogeneity of study outcomes and small number of programs that evaluated the health outcomes of funded projects, but qualitative data does provide evidence for key program components.

Nearly half of included studies report using an existing framework or model to guide community granting program development and implementation. There were examples of programs that used a framework or model reporting positive community and sustainability outcomes. The use of a framework or model may help guide the development of a granting program or community initiative and improve community mobilization and sustainability. Implementation science research supports the value of using frameworks and models in developing and implementing programs [82, 83]. Lack of theoretical guidance for design, implementation and evaluation of public health initiatives may contribute to a lack of sustainability of the funded community initiatives [84]. In this review, the most frequently cited framework or model was the Socioecological Model [80], cited by four granting programs. This model considers the interaction of four levels to impact health: individual, relationship, community and societal, reinforcing the critical role of social and structural determinants of health [85]. A socioecological approach is well-suited to designing strategies for community health improvement, as it provides a systems-oriented perspective to addressing unique health challenges of the community [16].

Using an evidence-informed approach to planning funded projects can help improve community health outcomes. Evidence used to inform projects should include data from the community as well as from the best available research literature [7]. In this review, only seven programs required that project proposals were informed by evidence. Two focused on community evidence, where community data was used to establish need, and five focused on published research evidence for effective interventions. These programs demonstrated an association between requiring an evidence-informed proposal and reporting positive health and community outcomes. There were several different sources of evidence used by programs in included studies, such as the Community Preventive Services Task Force’s Community Guide to Preventive Services Creating or Improving Places for Physical Activity and the National Cancer Institute’s Research Tested Intervention Programs database. Finding, using and applying evidence is inherently challenging for inexperienced community members, but community granting programs can help overcome this challenge by providing training and/or technical assistance and connecting applicants with pre-appraised, synthesized, and translated evidence. There are other sources of trustworthy evidence for interventions, such as the Health Evidence™ database [86], Health Systems Evidence [87], the World Health Organization’s guidelines [88], or the What Works for Health database [89].

There were several key components for community mobilization through community granting programs, including technical assistance and training, networking opportunities within the program, and skill-building for subsequent grant applications to promote sustainability of projects. Technical assistance and training were the most common program components described in studies, and were implemented in various ways, such as regular or ad hoc, and for a variety of topics for program implementation and other skills. Technical assistance and training are both common implementation strategies, shown to build the capacity of individuals to implement an intervention [90]. While most implementation strategies are provided to professionals, it is especially important to provide technical assistance and training to community members who may not have the relevant knowledge and skills to develop and implement a health-focused community project. Networking opportunities between awardees were also considered highly valuable. This aligns with evidence supporting peer-led learning as an effective strategy for adult learning [91–94].

Barriers to the completion of funded projects included timelines and the brevity of funding periods, noting difficulties in spending the full award in the allowed time. This aligns with the findings of the study that compared two models for community granting programs: one administered through a state health department, and another administered through an academic research organization [15]. This study found that a particular limitation within government-run models were the funding structures, which contributed to inflexible time lines and rigid accounting and reporting requirements [15]. Study authors note that frustrations due to funding negatively impacted the relationships between program staff and awardees [15]. The findings of this review reinforce the need for granting programs to be designed to be flexible to adequately meet the needs of community members and community-based organizations.

Finally, training for grant writing enhanced sustainability. While funding in included studies was time-limited, grant-writing training supported some awardees to secure additional funds. Several programs reported having secured additional funding, and awardees noted the value of training in grant writing for sustainability. Investment in this training can likely have long term effects on awardees’ impact on their communities.

Studies describe projects funded by community granting programs that were designed to meet the needs of populations that experience health inequities within their communities. These include programs designed for minority youth, Latinx communities and low-income populations [48, 52, 55]. Members of the community and community-based organizations are uniquely suited to develop projects that meet the needs of these communities [11]. Community-driven projects mobilize the community in driving their own health outcomes, by responding to needs and building on the strengths of those communities [12]. While only a handful of studies included in this review reported on community-level outcomes, those studies report positive impacts on social cohesion and community engagement in health promotion [45, 51, 63]. The positive impact on communities and numerous examples of engagement of populations subject to inequities reinforces the potential for community granting programs as a tool to empower these communities in reducing inequities.

A limitation of this review is that most of the included studies did not report outcomes on program goals for community mobilization and therefore an analysis of the relative contribution of various program components to community mobilization was limited. Future reporting of community mobilization targets, in addition to program outcomes, will enable a more robust analysis of the effect of community granting program components. Another limitation is that most studies did not report whether funded projects impacted community health outcomes. This is likely due to the challenges of data collection for awardees, who were often members of the community without experience in evaluation for health outcomes. Timelines for data collection were also likely a factor, as it may be difficult to measure health outcomes within a granting term. Future community granting programs may consider providing training for awardees in evaluation, providing additional funding for evaluation activities or evaluation experts, or requiring that awardees collect and report, however, the feasibility and additional administrative burden on awardees must be considered. Conducting this review as a rapid systematic review may increase the risk of bias in the review findings. The review was completed within a rapid timeline to inform the development of a provincial community granting program in Canada. Modifications to the full systematic review approach include using a single screener to determine eligibility of retrieved studies, and not blinding the second review to data extraction and quality assessment completed by the first reviewer. The impact of these modifications on potential bias in the review are likely minimal, given the efforts made to minimize potential bias, which included piloting a subset of references for screening and data extraction prior to completion by a single reviewer.

## Conclusion

This review provides a comprehensive overview and synthesis of studies of health-related community granting programs. The use of frameworks to guide program development supports a foundation for program success, by considering the various structural influences on community health. Grant awardees benefit from technical assistance, training, and networking opportunities for shared learning, and the sustainability of projects is enhanced by providing grant-writing support to awardees. Findings reinforce the potential for community granting programs to empower community-driven health promotion and improve community health outcomes. Several key components for granting program implementation were apparent, including guiding frameworks, providing technical assistance and training, networking opportunities for awardees, and skill-building for grant writing. There are fewer examples of community granting programs taking an evidence-informed approach to project selection and planning, but included studies provide insights into implementing evidence requirements for applicants. Overall, community granting programs can be a valuable strategy to drive community health outcomes, with several key elements supporting their success.

### Supplementary Information

**Supplementary Material 1.**

**Supplementary Material 2.**

## Data Availability

All data supporting the findings of this study are available within the paper and its Supplementary Information.

## Abbreviations

GRADE: Grading of Recommendations, Assessment, Development and Evaluations
HIV: Human immunodeficiency virus
JBI: Joanna Briggs Institute
PRISMA: Preferred Reporting Items for Systematic Reviews and Meta-Analyses
