# Supplementary material for: Mobilizing community-driven health promotion through community granting programs: a rapid systematic review
Source: BMC Public Health. 2024 Apr 1;24:932. doi: 10.1186/s12889-024-18443-8 (PMC10983705; doi:10.1186/s12889-024-18443-8)
Supplement: Supplementary file 1 — Supplementary Material 1. [file 12889_2024_18443_MOESM1_ESM.docx]

**Appendix 1: Search Strategy**

On March 16, 2023, the following databases were searched using the search terms listed.

Database: Ovid MEDLINE <1946 to 2023 March 06>

| # | Query |
| --- | --- |
| 1 | Financing, Organized/ or Financing, Government/ or Health Planning Support/ or Training Support/ or Research Support as Topic/ |
| 2 | (grant or grants or (grant* adj3 fund*) or endowment$ or mini-grant$ or minigrant$ or block grant$ or seed grant$ or seed-grant$ or (((endowment$ or grant$ or financial or funding or subsidy or subsidies or stipend$) adj2 (application$ or apply* or proposal$ or submission$ or submit* or requested or requesting)) or ((grant$ or endowment$ or subsidy or subsidies or stipend$) adj2 (allocat* or awardee or awards or awarded or criteria or decision* or panel or recipient* or rule$ or requirement$ or regulation$)) or (funding adj2 (academ* or communit* or decision* or research or project*)) or ((granted or granting or grants) adj2 (cash or financ* or funds or money or monetary))) or ((apply or applying or application$ or award* or grant or grants or granting) adj3 (cash or funds or funding or financ* or money or monetary or income or subsidy or subsidies or stipend$)) or ((agency or agencies or charity or charities or civic or civilian$ or department* or government* or group$ or federal* or foundation$ or ministry* or ministries or ministerial or national or "not for profit" or non profit or project$ or provinc* or public or region* or research or rural or state or territor* or tribe or tribal or urban) adj3 (endowment$ or grant or grants or stipend$))).ti,kw,kf,hw. |
| 3 | ((activit* or capital or child* or cultur* or hous* or equipment or environment* or facility or facilities or health or operating or pass through or philanthrop* or planning or playground$ or program* or project$ or recreation* or school$ or seed or senior$ or service* or social or sport* or start* up or start-up or support or technical or transport* or wellness or youth) adj3 (endowment$ or grant or grants or stipend$)).ti,kw,kf,hw. |
| 4 | (grant or grants or (grant* adj3 fund*) or endowment$ or mini-grant$ or minigrant$ or block grant$ or seed grant$ or seed-grant$ or (((endowment$ or grant$ or financial or funding or subsidy or subsidies or stipend$) adj2 (application$ or apply* or proposal$ or submission$ or submit* or requested or requesting)) or ((grant$ or endowment$ or subsidy or subsidies or stipend$) adj2 (allocat* or awardee or awards or awarded or criteria or decision* or panel or recipient* or rule$ or requirement$ or regulation$)) or (funding adj2 (academ* or communit* or decision* or research or project*)) or ((granted or granting or grants) adj2 (cash or financ* or funds or money or monetary))) or ((apply or applying or application$ or award* or grant or grants or granting) adj3 (cash or funds or funding or financ* or money or monetary or income or subsidy or subsidies or stipend$)) or ((agency or agencies or charity or charities or civic or civilian$ or department* or government* or group$ or federal* or foundation$ or ministry* or ministries or ministerial or national or "not for profit" or non profit or project$ or provinc* or public or region* or research or rural or state or territor* or tribe or tribal or urban) adj3 (endowment$ or grant or grants or stipend$))).ab. /freq=2 |
| 5 | ((activit* or capital or child* or cultur* or hous* or equipment or environment* or facility or facilities or health or operating or pass through or philanthrop* or planning or playground$ or program* or project$ or recreation* or school$ or seed or senior$ or service* or social or sport* or start* up or start-up or support or technical or transport* or wellness or youth) adj3 (endowment$ or grant or grants or stipend$)).ab. /freq=2 |
| 6 | ((granting or granted by or endowment from) adj3 (agency or agencies or body or bodies or foundation$ or organi$ation$)).mp. |
| 7 | or/1-6 |
| 8 | Program Evaluation/ or Program Development/ or Government Programs/ or Financing, Organized/mt, og, st, sn or Financing, Government/mt, og, st, sn or Health Planning Support/mt, og, st, sn or Research Support as Topic/mt, og, st, sn or methods/ or "organization and administration"/ or (mt or og).fs. or (administrat* or organiz* or organis* or manag* or program*).ti,hw. or (program* adj2 (aid or assistance or endowment or federal* or government* or grant$ or granting or ministerial or ministry or national or philanthrop* or provinc* or state$)).ti,kw,kf,hw. or ((assess* or administrat* or criteria or evaluat* or implement* or manag*) adj3 program*).ti,kw,kf,hw. |
| 9 | 7 and 8 |
| 10 | ((grant or grants or (grant* adj3 fund*) or endowment$ or funding or mini-grant$ or minigrant$ or block grant$ or seed grant$) adj3 (administrat* or criteria or evaluat* or implement* or initiative* or organiz* or organis* or manag* or program*)).ti,kw,kf,hw. or ((grant or grants or (grant* adj3 fund*) or endowment$ or mini-grant$ or minigrant$ or block grant$) adj1 (administrat* or initiative* or organiz* or organis* or manag* or program*)).ab. |
| 11 | 9 or 10 |
| 12 | Residence Characteristics/ or Community Networks/ or community-institutional relations/ |
| 13 | Cities/ or Community-Based Participatory Research/ or community resources/ or Local Government/ or rural health/ or Rural Health Services/ or Rural Population/ or suburban health/ or urban health/ or Urban Population/ or Urban Renewal/ or (city or cities or community or communities or district$ or hamlet or local* or municipal* or neighbour* or neighbor* or parish* or rural or suburban or town* or urban or village$).ti,kw,kf,hw. or (city or cities or community or communities or district$ or hamlet or local* or municipal* or neighbour* or neighbor* or parish* or rural or suburban or town* or urban or village$).ab. /freq=2 |
| 14 | ((city* or cities or civilian$ or civic or community or communities or district$ or hamlet$ or local or municipal* or neighbour* or neighbor* or parish* or regional or resident$ or residenc* or town* or village$) adj1 (agency or agencies or alliance$ or applicant$ or application$ or association$ or auxilliar* or club$ or coalition$ or collective$ or co-operative$ or committee$ or communit* or council or effort$ or facilit* or facility or facilities or federation$ or foundation$ or initiative$ or group$ or guild or league$ or led or leadership or lead or organization$ or organisation$ or program* or project$ or propos* or submit* or submission$ or service* or society or societies or team*)).mp. |
| 15 | charities/ or foundations/ or organizations/ or organizations, nonprofit/ or population groups/ or exp self-help groups/ or social group/ or societies/ or mental health associations/ or alcoholics anonymous/ or american speech-language-hearing association/ or societies, scientific/ or health planning councils/ or hospital auxiliaries/ or "state health planning and development agencies"/ or health systems agencies/ or health care coalitions/ or home care agencies/ or voluntary health agencies/ or "college fraternities and sororities"/ or faith-based organizations/ or Organizational Affiliation/ or Organizational Objectives/ or Efficiency, Organizational/ or Organizational Culture/ or Models, Organizational/ or Organizational Innovation/ or community networks/ or social structure/ or social group/ or Social Network Analysis/ or group processes/ or group dynamics/ or group structure/ [groups 1] |
| 16 | Fitness centers/ or "Sports and Recreational Facilities"/ or child day care centers/ or public facilities/ or assisted living facilities/ or group homes/ or halfway houses/ or homes for the aged/ or orphanages/ or senior centers/ or adult day care centers/ or exp "sports and recreational facilities"/ or exp rehabilitation centers/ or exp residential facilities/ [association defined by community space] |
| 17 | ("ethnic and racial minorities"/ or exp "health disparate, minority and vulnerable populations"/ or exp indigenous peoples/ or exp population groups/ or exp students/ or exp survivors/ or "transients and migrants"/ or veterans/ or volunteers/ or minority groups/ or social class/ or exp racial groups/ or refugees/ or exp "emigrants and immigrants"/ or african americans/ or amish/ or arabs/ or asian americans/ or jews/ or roma/ or exp "Sexual and Gender Minorities"/ or Vulnerable Populations/ or "transients and migrants"/ or *Students/ or alcoholics/ or exp disabled persons/ or disaster victims/ or drug users/ or exp homeless persons/ or pedestrians/ or students/ or survivors/ or veterans/ or exp volunteers/ or working poor/ or Workplace/ or gardens/ or exp art/ or exp culture/ or exp literature/ or exp music/ or religion/ or buddhism/ or exp christianity/ or hinduism/ or islam/ or judaism/ or social change/ or social responsibility/ or social planning/ or social welfare/ or Environment/ or horticulture/ or Agriculture/) and (committee membership/ or Cooperative Behavior/ or organizations/ or organizations, nonprofit/ or public-private sector partnerships/ or societies/ or community networks/ or community support/ or collective efficacy/ or cooperative behavior/ or Group Dynamics/ or group processes/ or leadership/ or Psychology, Social/ or Psychosocial Support Systems/ or social behavior/ or social integration/ or social cohesion/ or social identification/ or social inclusion/ or sociology/ or social planning/ or social structure/ or social group/ or social capital/ or social environment/ or social support/ or Social Networking/ or Stakeholder Participation/ or (advocacy or agency or agencies or alliance$ or association$ or collective* or community or communities or cooperat* or co-operat* or group$ or guild$ or collective* or collaborat* or league$ or organization or organisation or partnership$ or support*).ti,kw,kf,hw.) [population segment and organizing principle] |
| 18 | (charities or communit* of practice or societies or ((activis* or adolescent or advoca* or agricultur* or art$ or artist* or artisan$ or anti* or advocacy or athlet* or boys or girls or business or charity or charities or children$ or church or citizen$ or civic or civillian or cultur* or development* or disabil* or disable* or elder$ or employee$ or environment* or ethnic* or faith-based or first-nation$ or fitness or food or gay$ or heritage* or home* or immigrant or indigenous or inter-faith or inter-cultur* or fitness or (health adj2 promot*) or intramural or intra-mural or lesbian$ or LGBTQ or local or men$ or mans or minority or municipal* or neighbourhood or neighborhood or non-government* or non-profit or "not for profit" or nutrition* or outdoor$ or parent* or partnership$ or poverty or recreation* or religious or rural or service* or school$ or senior$ or social* or society or sport* or student$ or support* or teach* or teen* or transport* or tribal or urban or volunteer* or vulnerable or wellness or women or woman$ or worker$ or workplace$ or youth) adj1 (agency or agencies or alliance$ or association$ or auxilliar* or club$ or coalition$ or collective$ or collaborat* or cooperat* or co-operative$ or committee$ or communit* or council or effort$ or facilit* or federation$ or foundation$ or group$ or guild or facility or facilities or initiative$ or league$ or led or leadership or lead or organization$ or organisation$ or partner* or program* or project$ or service* or society or societies or team*))).ti,kw,kf. [kw for community groups] |
| 19 | or/12-18 |
| 20 | 11 and 19 |
| 21 | ((civic or communit* or district$ or local* or municipal* or neighbour or neighbor* or residenc* or resident* or rural* or suburban* or tribe$ or tribal or urban*) adj3 (block grant$ or grant$ or endowment$ or minigrant* or mini-grant$ or seed grant$)).ti,kw,kf,hw. |
| 22 | ((agency or agencies or auxiliaries or club or clubs or collective$ or cooperat* or co-operat* or facility or facilities or foundation$ or group or groups or guild$ or league$ or organisation or organisations or organization or organizations or societies or society or team$) adj3 ("block grant*" or grant or grants or endowment* or minigrant or mini-grant or "mini grant" or "seed grant*")).ti,kw,kf,hw. |
| 23 | 20 or 21 or 22 |
| 24 | limit 23 to yr="2008 -Current" |

Database: SocINDEX <2008 to 2023 December 31>

| # | Query |
| --- | --- |
|  | Filtered to academic journals and conference papers |
| S31 | S29 AND S30 |
| S30 |  |
| S29 | S26 OR S27 OR S28 |
| S28 | TI ( ((agency or agencies or auxiliaries or club or clubs or collective* or cooperat* or co-operat* or facility or facilities or foundation* or group or groups or guild* or league* or organisation or organisations or organization or organizations or society or societies or team or teams) N3 ( ("block grant*" or grant or granted or grants or endowment* or minigrant or mini-grant or "mini grant" or "seed grant*")) ) OR KW ( ((agency or agencies or auxiliaries or club or clubs or collective* or cooperat* or co-operat* or facility or facilities or foundation* or group or groups or guild* or league* or organisation or organisations or organization or organizations or society or societies or team or teams) N3 ( ("block grant*" or grant or granted or grants or endowment* or minigrant or mini-grant or "mini grant" or "seed grant*")) ) OR DE ( ((agency or agencies or auxiliaries or club or clubs or collective* or cooperat* or co-operat* or facility or facilities or foundation* or group or groups or guild* or league* or organisation or organisations or organization or organizations or society or societies or team or teams) N3 ( ("block grant*" or grant or granted or grants or endowment* or minigrant or mini-grant or "mini grant" or "seed grant*")) ) |
| S27 | TI ( ((civic or communit* or district* or local* or municipal* or neighbour* or neighbour* or residenc* or resident* or rural* or suburban* or tribe* or tribal or urban*) N3 ("block grant*" or grant* or endowment* or minigrant or mini-grant or "mini grant" or "seed grant*")) ) OR KW ( ((civic or communit* or district* or local* or municipal* or neighbour* or neighbour* or residenc* or resident* or rural* or suburban* or tribe* or tribal or urban*) N3 ("block grant*" or grant* or endowment* or minigrant or mini-grant or "mini grant" or "seed grant*")) ) or SU ((civic or communit* or district* or local* or municipal* or neighbour* or neighbour* or residenc* or resident* or rural* or suburban* or tribe* or tribal or urban*) N3 ("block grant*" or grant* or endowment* or minigrant or mini-grant or "mini grant" or "seed grant*")) OR DE ((civic or communit* or district* or local* or municipal* or neighbour* or neighbour* or residenc* or resident* or rural* or suburban* or tribe* or tribal or urban*) N3 ("block grant*" or grant* or endowment* or minigrant or mini-grant or "mini grant" or "seed grant*")) |
| S26 | S14 AND S25 |
| S25 | S16 OR S17 OR S18 OR S19 OR S20 OR S21 OR S24 |
| S24 | S22 AND S23 |
| S23 | ( (DE "MINORITIES" OR DE "CHILDREN of minorities" OR DE "CHURCH & minorities" OR DE "DISABLED minorities" OR DE "LINGUISTIC minorities" OR DE "MINORITY LGBTQ people" OR DE "MINORITY boys" OR DE "MINORITY families" OR DE "MINORITY gangs" OR DE "MINORITY girls" OR DE "MINORITY older people" OR DE "MINORITY parents" OR DE "MINORITY students" OR DE "MINORITY teachers" OR DE "MINORITY women" OR DE "MINORITY youth" OR DE "RACIAL minorities" OR DE "RELIGIOUS minorities" OR DE "SEXUAL minorities" OR DE "URBAN minorities" OR DE "PEOPLE of color") OR (DE "MIDDLE class" OR DE "UPPER class" OR DE "CLASS identity" OR DE "POOR people" OR DE "WORKING class")) OR (DE "SOCIAL classes" OR DE "POOR people" OR DE "UNDERCLASS")) OR (DE "ETHNICITY" OR DE "ETHNIC identity of African American women" OR DE "ETHNIC identity of Africans" OR DE "ETHNIC identity of Amerasians" OR DE "ETHNIC identity of Arab Americans" OR DE "ETHNIC groups")) OR (DE "REFUGEES" OR DE "ENVIRONMENTAL refugees" OR DE "POLITICAL refugees" OR DE "REFUGEE children") ) OR ( (DE "PEOPLE with alcoholism")) OR (DE "PEOPLE with disabilities")) OR (DE "VICTIMS")) OR (DE "PEOPLE with drug addiction")) OR (DE "ROGUES & vagabonds")) OR DE "TRAMPS")) OR (DE "VETERANS")) OR (DE "VOLUNTEERS")) OR (DE "WORKING poor") ) OR DE "INDIGENOUS ethnic identity" OR DE "INDIGENOUS peoples" OR DE "INDIGENOUS children" OR DE "INDIGENOUS youth" OR DE "SOCIAL work with indigenous peoples" OR DE "BLACK race" OR DE "RACIAL minorities" OR DE "ETHNICITY" OR DE "ETHNIC identity of African American women" OR DE "ETHNIC identity of Africans" OR DE "ETHNIC identity of Amerasians" OR DE "ETHNIC identity of Arab Americans" OR DE "ETHNIC identity of Arabs" OR DE "ETHNIC identity of Armenian Americans" OR DE "ETHNIC identity of Asian Americans" OR DE "ETHNIC identity of Berbers" OR DE "ETHNIC identity of British people" OR DE "ETHNIC identity of Canadians" OR DE "ETHNIC identity of Cherokee Indians" OR DE "ETHNIC identity of Chinese" OR DE "ETHNIC identity of Chinese Canadians" OR DE "ETHNIC identity of Creoles" OR DE "ETHNIC identity of Cuban Americans" OR DE "ETHNIC identity of Dominican Americans" OR DE "ETHNIC identity of Dutch people" OR DE "ETHNIC identity of French Americans" OR DE "ETHNIC identity of French-Canadians" OR DE "ETHNIC identity of German Americans" OR DE "ETHNIC identity of Germans" OR DE "ETHNIC identity of Greeks" OR DE "ETHNIC identity of Hawaiians" OR DE "ETHNIC identity of Hispanic Americans" OR DE "ETHNIC identity of Indian Americans" OR DE "ETHNIC identity of Italian Americans" OR DE "ETHNIC identity of Korean Americans" OR DE "ETHNIC identity of Latin Americans" OR DE "ETHNIC identity of Maya Indians" OR DE "ETHNIC identity of Mexicans" OR DE "ETHNIC identity of Pacific Islanders" OR DE "ETHNIC identity of Polish Americans" OR DE "ETHNIC identity of Puerto Ricans" OR DE "ETHNIC identity of Romanies" OR DE "ETHNIC identity of Scots" OR DE "ETHNIC identity of South Asians" OR DE "ETHNIC identity of Welsh" OR DE "ETHNICITY in children" OR DE "ETHNICITY in women" OR DE "INDIGENOUS ethnic identity" OR DE "IRISH people -- Ethnic identity" OR DE "MAORI ethnic identity" OR DE "MULTIGROUP Ethnic Identity Measure" OR DE "MULTIRACIAL identity" OR DE "RACIAL identity of black people" OR DE "RACIAL identity of whites" OR DE "UKRAINIANS -- Ethnic identity" OR DE "RACE" OR DE "RELIGIONS" OR DE "ASSYRO-Babylonian religion" OR DE "BRAHMANISM" OR DE "BUDDHISM" OR DE "CONFUCIANISM" OR DE "CULTS" OR DE "DRUIDS & druidism" OR DE "HINDUISM" OR DE "JAINISM" OR DE "MYSTERY religions" OR DE "MYTHOLOGY" OR DE "NEO-Confucianism" OR DE "NEOPAGANISM" OR DE "OCCULTISM" OR DE "PAGANISM" OR DE "POLYTHEISM" OR DE "POSITIVISM" OR DE "RELIGIONS (Proposed, universal, etc.)" OR DE "RELIGIOUS denominations" OR DE "RELIGIOUS diversity" OR DE "SECTS" OR DE "SHAMANISM" OR DE "SHINTO" OR DE "SIKHISM" OR DE "SYNCRETISM (Religion)" OR DE "TAOISM" OR DE "ZOROASTRIANISM" OR DE "RELIGION" OR DE "RELIGIOUS groups" OR DE "RELIGIOUS institutions") OR (DE "CULTURAL identity" OR DE "JEWISH identity" OR DE "LINGUISTIC identity" OR DE "ORGANIZATIONAL identification" OR DE "RACE identity" OR DE "AFFILIATION (Psychology)" OR DE "REFERENCE groups") OR (DE "ASCETICS" OR DE "BAHAIS" OR DE "BUDDHISTS" OR DE "CHRISTIANS" OR DE "CONFUCIANISTS" OR DE "GENTILES" OR DE "HINDUS" OR DE "JAINS" OR DE "JEWS" OR DE "MUSLIMS" OR DE "SHINTOISTS" OR DE "SIKHS" OR DE "TAOISTS" OR DE "ZOROASTRIANS") OR (DE "STUDENTS")) OR (((DE "PEOPLE with alcoholism" OR DE "PEOPLE with addiction") OR (DE "TRANSGENDER people" OR DE "GENDER-nonconforming people" OR DE "BIGENDER people" OR DE "INTERSEX people" OR DE "MULTIRACIAL identity")) OR (DE "VICTIMS" OR DE "VOLUNTEERS")) OR (DE "WORK environment") OR (DE "SPORTS participation" OR DE "SCHOOL sports")) OR (DE "ART" OR DE "STREET art" OR DE "MUSIC" OR DE "ART & culture" OR DE "ARTS & society" OR DE "ARTS" OR DE "ARTISTS" OR DE "URBAN beautification" OR DE "DANCE" OR DE "CULTURAL movements" OR DE "CULTURAL activities")) OR (((((((((DE "PARKS" OR DE "COMMONS" OR DE "RECREATION areas" OR DE "URBAN parks" OR DE "PUBLIC spaces") OR (DE "SOCIAL change")) OR (DE "SOCIAL responsibility")) OR (DE "SOCIAL planning")) OR (DE "COMMUNITY development")) OR (DE "ECONOMIC development")) OR (DE "ECONOMIC development projects")) OR (DE "COMMON good")) OR (DE "ENVIRONMENTAL policy" OR DE "ECOLOGY" OR DE "URBAN land use" OR DE "URBAN planning" OR DE "URBAN planning & the environment")) OR (DE "SUSTAINABLE agriculture" OR DE "URBAN agriculture" OR DE "COMMUNITY-supported agriculture") OR (DE "NATURE conservation") OR (DE "ECONOMIC development projects" OR DE "RURAL development projects")) |
| S22 | ( (((((((((((((((((((((((((((((((DE "CITIZENS' advisory committees" OR DE "ADVISORY boards" OR DE "CITIZENS' associations" OR DE "COOPERATION") OR (DE "COOPERATIVE societies")) OR (DE "INSTITUTIONAL cooperation")) OR (DE "COOPERATIVENESS")) OR (DE "REGIONAL cooperation" OR DE "SHARED leadership" OR DE "LEADERSHIP" OR DE "COMMUNITY leadership")) OR (DE "GROUPS")) OR (DE "ACTIVISM" OR DE "ASSOCIATIONS, institutions, etc." OR DE "ACTIVISTS")) OR (DE "ORGANIZATION" OR DE "COMMUNITY organization" OR DE "ORGANIZATIONAL research")) OR (DE "STAKEHOLDER theory" OR DE "GRASSROOTS movements" OR DE "HUMAN capital")) OR (DE "GROUP dynamics" OR DE "GROUP process" OR DE "GROUP formation")) OR (DE "SOCIETIES" OR DE "COOPERATIVE societies")) OR (DE "COMMUNITY development")) OR (DE "COLLECTIVES" OR DE "COLLECTIVE action" OR DE "COLLECTIVES (Social movements)" OR DE "GROUP identity" OR DE "COLLECTIVE behavior"))) OR (DE "SOCIAL psychology")) OR (DE "SOCIAL support" OR DE "SOCIAL networks")) OR (DE "SOCIAL integration" OR DE "SOCIAL belonging")) OR (DE "SOCIAL cohesion")) OR (DE "SOLIDARITY" OR DE "SOCIAL structure")) OR (DE "SOCIOLOGY")) OR (DE "INSTITUTION building" OR DE "SOCIAL institutions")) OR (DE "SOCIAL belonging")) OR (DE "EMBEDDEDNESS (Socioeconomic theory)")) OR (DE "SOCIAL bonds")) OR (DE "SOCIAL processes")) OR (DE "ORGANIZATIONAL commitment")) OR (DE "ORGANIZATIONAL identification" OR DE "ORGANIZATIONAL socialization")) OR (DE "COMMUNITY development" OR DE "SOCIAL planning")) OR (DE "SOCIAL structure" OR DE "SOCIAL group work" OR DE "SOCIAL groups")) OR (DE "SOCIAL capital")) OR (DE "SOCIAL conditioning" OR DE "SOCIAL context")) OR (DE "SOCIAL conditioning") OR (DE "COOPERATIVE societies" OR DE "PRODUCER cooperatives" OR DE "COOPERATION" OR DE "INTELLECTUAL cooperation") OR (DE "COOPERATIVENESS")) OR (DE "COOPERATION & socialism")) OR (DE "ASSOCIATIONS, institutions, etc." OR DE "SOCIETIES" OR DE "COLLECTIVES" OR DE "COOPERATION" OR DE "SOCIAL movements")) OR (DE "GUILDS")) OR (DE "NONPROFIT organizations" OR DE "NONGOVERNMENTAL organizations") ) ) OR ( TI ( (advocacy or agency or agencies or alliance* or application* or applicant or association* or collective* or community or communities or collaboration or group or groups or league or partner* or support*) ) OR KW ( (advocacy or agency or agencies or alliance* or application* or applicant or association* or collective* or community or communities or collaboration or group or groups or league or partner* or support*) ) OR ( DE (advocacy or agency or agencies or alliance* or application* or applicant or association* or collective* or community or communities or collaboration or group or groups or league or partner* or support*) ) ) ) ) |
| S21 | TI ( (charities or "communit* of practice" or societies or ((activis* or adolescent or advoca* or agricultur* or art or arts or artist* or artisan* or anti* or advocacy or athlet* or boys or girls or business or charity or charities or children* or church or citizen or citizens or civic or civillian or cultur* or development* or disabil* or disable* or elder* or employee* or environment* or ethnic* or faith-based or first-nation* or fitness or food or gay or heritage* or home* or immigrant or indigenous or inter-faith or inter-cultur* or fitness or (health N2 promot*) or intramural or intra-mural or lesbian* or LGBTQ or local or men or men's or man or man's or minority or municipal* or neighbourhood or neighborhood or non-government* or non-profit or "not for profit" or nutrition* or outdoor* or parent* or partnership* or poverty or recreation* or religious or rural or service* or school* or senior* or social* or society or sport* or student* or support* or teach* or teen* or transport* or tribal or urban or volunteer* or vulnerable or wellness or women or woman* or worker* or workplace* or youth) adj1 (agency or agencies or alliance* or association* or auxilliar* or club or clubs or coalition* or collective* or collaborat* or cooperat* or co-operative* or committee* or communit* or council or councils or effort or efforts or facilit* or federation* or foundation* or group or groups or guild or guilds or facility or facilities or initiative* or league* or led or leadership or lead or organization* or organisation* or partner* or program* or project* or service* or society or societies or team*)) ) OR KW ( (charities or "communit* of practice" or societies or ((activis* or adolescent or advoca* or agricultur* or art or arts or artist* or artisan* or anti* or advocacy or athlet* or boys or girls or business or charity or charities or children* or church or citizen or citizens or civic or civillian or cultur* or development* or disabil* or disable* or elder* or employee* or environment* or ethnic* or faith-based or first-nation* or fitness or food or gay or heritage* or home* or immigrant or indigenous or inter-faith or inter-cultur* or fitness or (health N2 promot*) or intramural or intra-mural or lesbian* or LGBTQ or local or men or men's or man or man's or minority or municipal* or neighbourhood or neighborhood or non-government* or non-profit or "not for profit" or nutrition* or outdoor* or parent* or partnership* or poverty or recreation* or religious or rural or service* or school* or senior* or social* or society or sport* or student* or support* or teach* or teen* or transport* or tribal or urban or volunteer* or vulnerable or wellness or women or woman* or worker* or workplace* or youth) adj1 (agency or agencies or alliance* or association* or auxilliar* or club or clubs or coalition* or collective* or collaborat* or cooperat* or co-operative* or committee* or communit* or council or councils or effort or efforts or facilit* or federation* or foundation* or group or groups or guild or guilds or facility or facilities or initiative* or league* or led or leadership or lead or organization* or organisation* or partner* or program* or project* or service* or society or societies or team*)) ) OR ( DE (charities or "communit* of practice" or societies or ((activis* or adolescent or advoca* or agricultur* or art or arts or artist* or artisan* or anti* or advocacy or athlet* or boys or girls or business or charity or charities or children* or church or citizen or citizens or civic or civillian or cultur* or development* or disabil* or disable* or elder* or employee* or environment* or ethnic* or faith-based or first-nation* or fitness or food or gay or heritage* or home* or immigrant or indigenous or inter-faith or inter-cultur* or fitness or (health N2 promot*) or intramural or intra-mural or lesbian* or LGBTQ or local or men or men's or man or man's or minority or municipal* or neighbourhood or neighborhood or non-government* or non-profit or "not for profit" or nutrition* or outdoor* or parent* or partnership* or poverty or recreation* or religious or rural or service* or school* or senior* or social* or society or sport* or student* or support* or teach* or teen* or transport* or tribal or urban or volunteer* or vulnerable or wellness or women or woman* or worker* or workplace* or youth) adj1 (agency or agencies or alliance* or association* or auxilliar* or club or clubs or coalition* or collective* or collaborat* or cooperat* or co-operative* or committee* or communit* or council or councils or effort or efforts or facilit* or federation* or foundation* or group or groups or guild or guilds or facility or facilities or initiative* or league* or led or leadership or lead or organization* or organisation* or partner* or program* or project* or service* or society or societies or team*)) ) |
| S20 | (((((((((((DE "COMMUNITY centers" OR DE "DAY care centers" OR DE "RECREATION centers" OR DE "LGBTQ community centers" OR DE "DAY care centers for people with disabilities" OR DE "DAY care centers for people with intellectual disabilities" OR DE "YOUTH centers" OR DE "CRISIS centers" OR DE "CONGREGATE housing") OR (DE "GROUP homes")) OR (DE "ALMSHOUSES")) OR (DE "INSTITUTIONAL care of older people")) OR (DE "DAY care centers for people with disabilities")) OR (DE "SCHOOL facilities")) OR (DE "HALFWAY houses")) OR (DE "SHARED housing")) OR (DE "CONGREGATE housing")) OR (DE "OUTPATIENT substance abuse treatment facilities")) OR (DE "OUTPATIENT mental health facilities")) OR (DE "LGBTQ community centers") OR (DE "SENIOR housing"))OR (DE "SOBER living environments")) OR (DE "INSTITUTIONAL care") OR TI ( center or centers or facility or facilities ) OR KW ( center or centers or facility or facilities ) OR ( DE (center or centers or facility or facilities) ) |
| S19 | ((((((((((((((DE "PARENTS' & teachers' associations" OR DE "ASSOCIATIONS, institutions, etc." OR DE "CITIZENS' associations" OR DE "MEMBERSHIP in associations, institutions, etc." OR DE "INSTITUTIONAL cooperation" OR DE "SPECIAL interest groups (Associations)" OR DE "SENIOR citizen's clubs" OR DE "GUILDS" OR DE "WOMEN'S societies & clubs" OR DE "SOCIETIES" OR DE "SOCIAL clubs" OR DE "SISTERHOODS" OR DE "BROTHERHOODS") OR (DE "AD hoc organizations")) OR (DE "NONGOVERNMENTAL organizations" OR DE "NONPROFIT organizations")) OR (DE "LGBTQ organizations" OR DE "HEALTH maintenance organizations" OR DE "RELIGIOUS institutions" OR DE "COMMUNITY organization" OR DE "LGBTQ student organizations & activities" OR DE "CIVIL rights organizations" OR DE "SOCIAL health maintenance organizations" OR DE "YOUTH societies & clubs" OR DE "SUPPORT groups")) OR (DE "ORGANIZATIONAL sociology" OR DE "ORGANIZATIONAL learning" OR DE "ORGANIZATIONAL behavior" OR DE "CHARITIES" OR DE "TWELVE-step programs")) OR (DE "SOCIETIES" OR DE "ORGANIZATIONAL power" OR DE "ORGANIZATIONAL goals" OR DE "ORGANIZATIONAL change")) OR (DE "NONPROFIT sector")) OR (DE "LEARNED institutions & societies" OR DE "INTERORGANIZATIONAL relations" OR DE "INTERORGANIZATIONAL networks" OR DE "GREEK letter societies" OR DE "GAY-straight alliances in schools" OR DE "FAITH-based initiative (Government program)" OR DE "ESTABLISHED churches")) OR (DE "FOLLOWERSHIP")) OR (DE "COOPERATIVE societies" OR DE "COMMUNITY involvement" OR DE "COLLECTIVES (Social movements)" OR DE "CHURCH societies" OR DE "CATHOLIC institutions")) OR (DE "COMMUNITY organization")) OR (DE "COALITIONS" OR DE "SOCIAL groups" OR DE "ALLIES (LGBTQ supporters)")) OR (DE "GROUP formation"))) OR (DE "SOCIAL action") OR (DE "TEAMS")) OR (DE "PARTNERSHIPS in education")) OR (DE "GROUPS")) OR (DE "PRESSURE groups" OR DE "RESEARCH institutes" OR DE "SOCIAL group work" OR (DE "VOLUNTEER service")) OR (DE "ARTISTS & community" OR DE "COMMUNITARIANISM" OR DE "COMMUNITY life" OR DE "COMMUNITY-based participatory research") OR ( DE "SOCIAL enterprises" OR DE "CHARITIES") OR (DE "SUPPORT groups for substance abusers" OR DE "SMALL groups" OR DE "REFERENCE groups" OR DE "SOCIAL groups research" OR DE "RELIGIOUS groups" OR DE "LGBTQ affinity groups" OR DE "HOMOSOCIAL groups" OR DE "AFFINITY groups" OR DE "AD hoc organizations" OR DE "SOCIAL group work" OR DE "SMALL group research" OR DE "GROUP rights" OR DE "RELIGIOUS communities" OR DE "PRIMARY groups (Social groups)" OR DE "CIVIL rights organizations" OR DE "TRIBES" OR DE "TEAMS") OR (DE "NONGOVERNMENTAL organizations")) OR (DE "RESEARCH institutes" OR DE "SOCIAL group work") OR (DE "SOCIAL groups" OR DE "VOLUNTEER service")) OR (DE "ORGANIZATIONAL change")) OR (DE "SOCIAL networks" OR DE "SOCIAL structure" OR DE "ORGANIZATIONAL sociology" OR DE "ORGANIZATIONAL ecology" OR DE "ORGANIZATIONAL identification" OR DE "ORGANIZATIONAL learning" OR DE "ORGANIZATIONAL behavior research" OR DE "SOCIAL structure" OR DE "ORGANIZATIONAL research" OR DE "ORGANIZATIONAL power" OR DE "ORGANIZATIONAL growth" OR DE "ORGANIZATIONAL effectiveness" OR DE "ORGANIZATIONAL citizenship behavior" OR DE "ORGANIZATIONAL behavior") |
| S18 | TI ((city* or cities or civillian* or civic or community or communities or district* or hamlet* or local or municipal* or neighbour* or neighbor* or parish* or regional or resident* or residenc* or town* or village*) N1 (agency or agencies or auxilliar* or club or clubs or coalition* or collective* or co-operative* or cooperative* or committee* or communit* or council* or effort* or facilities or facility or federation* or foundation* or initiative* or group or groups or guild or guilds or league* or led or leadership or lead or organization* or organisation* or program* or project* or propos* or submit* or submission* or service or society or societies or team*)) OR KW ((city* or cities or civillian* or civic or community or communities or district* or hamlet* or local or municipal* or neighbour* or neighbor* or parish* or regional or resident* or residenc* or town* or village*) N1 (agency or agencies or auxilliar* or club or clubs or coalition* or collective* or co-operative* or cooperative* or committee* or communit* or council* or effort* or facilities or facility or federation* or foundation* or initiative* or group or groups or guild or guilds or league* or led or leadership or lead or organization* or organisation* or program* or project* or propos* or submit* or submission* or service or society or societies or team*)) OR DE ((city* or cities or civillian* or civic or community or communities or district* or hamlet* or local or municipal* or neighbour* or neighbor* or parish* or regional or resident* or residenc* or town* or village*) N1 (agency or agencies or auxilliar* or club or clubs or coalition* or collective* or co-operative* or cooperative* or committee* or communit* or council* or effort* or facilities or facility or federation* or foundation* or initiative* or group or groups or guild or guilds or league* or led or leadership or lead or organization* or organisation* or program* or project* or propos* or submit* or submission* or service or society or societies or team*)) OR AB ((city* or cities or civillian* or civic or community or communities or district* or hamlet* or local or municipal* or neighbour* or neighbor* or parish* or regional or resident* or residenc* or town* or village*) N1 (agency or agencies or auxilliar* or club or clubs or coalition* or collective* or co-operative* or cooperative* or committee* or communit* or council* or effort* or facilities or facility or federation* or foundation* or initiative* or group or groups or guild or guilds or league* or led or leadership or lead or organization* or organisation* or program* or project* or propos* or submit* or submission* or service or society or societies or team*)) |
| S17 | ( TI ( (city or cities or community or communities or district* or hamlet or local* or municipal* or neighbour or neighbor* or rural* or parish or suburban* or town* or urban* or village) ) OR KW ( (city or cities or community or communities or district* or hamlet or local* or municipal* or neighbour or neighbor* or rural* or parish or suburban* or town* or urban* or village) ) OR DE ( (city or cities or community or communities or district* or hamlet or local* or municipal* or neighbour or neighbor* or rural* or parish or suburban* or town* or urban* or village) ) |
| S16 | (((((((DE "NEIGHBORHOODS" OR (DE "COMMUNITIES" OR DE "CITIES & towns" OR DE "HUMAN settlements" OR DE "RURAL sociology" OR DE "VILLAGE communities" OR DE "COMMUNITY centers" OR DE "COMMUNITY organization" OR DE "COMMUNITY size" OR DE "COMMUNITY-based participatory research" OR DE "SUBURBS")) OR (DE "INNER cities")) OR DE "URBAN sociology" OR DE "URBAN health")) OR (DE "SUBURBS")) OR (DE "RURAL health")) OR (DE "RURAL population")) OR (DE "CITY dwellers")) OR (DE "MUNICIPAL government") OR (DE "COMMUNITY welfare councils") OR (DE "CITY council members") OR (DE "COMMUNITY organization") OR (DE "COMMUNITY-based child welfare" OR DE "COMMUNITY-based social services")) OR DE ("COMMUNITY development" OR DE "URBAN community development") OR (DE "planned communities")OR (DE "SOCIAL settlements") OR DE (community or communities) OR TI (community or communities) |
| S15 | TI blank |
| S14 | S12 OR S13 |
| S13 | TI ((grant or grants or (grant N2 fund*) or endowment* or funding or mini-grant* or minigrant* or "block grant*" or "seed grant*" or seed-grant*) N3 (administrat* or criteria or evaluat* or implement* or initiative* or organiz* or organis* or manag* or program*)) OR SU ((grant or grants or (grant N2 fund*) or endowment* or funding or mini-grant* or minigrant* or "block grant*" or "seed grant*" or seed-grant*) N3 (administrat* or criteria or evaluat* or implement* or initiative* or organiz* or organis* or manag* or program*)) OR KW ((grant or grants or (grant N2 fund*) or endowment* or funding or mini-grant* or minigrant* or "block grant*" or "seed grant*" or seed-grant*) N3 (administrat* or criteria or evaluat* or implement* or initiative* or organiz* or organis* or manag* or program*)) OR AB ((grant or grants or (grant N2 fund*) or endowment* or funding or mini-grant* or minigrant* or "block grant*" or "seed grant*" or seed-grant*) N1 (administrat* or criteria or evaluat* or implement* or initiative* or organiz* or organis* or manag* or program*)) |
| S12 | S7 AND S11 |
| S11 | S8 OR S9 OR S10 |
| S10 | TI ((assess* or administrat* or criteria or evaluat* or implement* or manag*) N3 program*) ) OR SU ( ((assess* or administrat* or criteria or evaluat* or implement* or manag*) N3 program*) ) OR KW ( ((assess* or administrat* or criteria or evaluat* or implement* or manag*) N3 program*) ) OR ( DE ((assess* or administrat* or criteria or evaluat* or implement* or manag*) N3 program*) ) |
| S9 | ((((((DE "PUBLIC administration") OR (DE "MANAGEMENT of human services")) OR (DE "POLICY sciences")) OR (DE "POLITICAL planning")) OR (DE "GOVERNMENT policy")) OR TI (administrat* or organiz* or organis* or manag* or program*) or DE (administrat* or organiz* or organis* or manag* or program*) |
| S8 | (DE "GOVERNMENT programs") OR (DE "GOVERNMENT aid") OR (DE "EVALUATION research (Social action programs)" OR DE "EVALUATION of human services programs" OR (DE "EVALUATION of organizational effectiveness") OR (DE "OUTCOME assessment (Social services)") OR TI (program* N2 (aid or assistance endowment or federal* or grant or granting or government* or ministerial or ministry or national or philanthrop* or provinc* or state or states)) OR KW (program* N2 (aid or assistance endowment or federal* or grant or granting or government* or ministerial or ministry or national or philanthrop* or provinc* or state or states)) OR DE (program* N2 (aid or assistance endowment or federal* or grant or granting or government* or ministerial or ministry or national or philanthrop* or provinc* or state or states)) |
| S7 | S1 OR S2 OR S3 OR S4 OR S5 OR S6 |
| S6 | AB ((activit* or capital or hous* or equipment or environment* or facility or facilities or operating or "pass through" or philanthrop* or project or projects or recreation* or service* or "start* up" or start-up or support or technical) N1 (endowment* or grant or grants or stipend*)) |
| S5 | TI ((activit* or capital or child* or cultur* or health or hous* or equipment or environment* or facility or facilities or operating or "pass through" or philanthrop* or planning or playground* or program* or project or projects or recreation* or senior* or service* or social or sport or "start* up" or start-up or support or technical or transportation or wellness or youth) N3 (endowment* or grant or grants or stipend*)) OR KW ((activit* or capital or child* or cultur* or health or hous* or equipment or environment* or facility or facilities or operating or "pass through" or philanthrop* or planning or playground* or program* or project or projects or recreation* or senior* or service* or social or sport or "start* up" or start-up or support or technical or transportation or wellness or youth) N3 (endowment* or grant or grants or stipend*)) OR SU ((activit* or capital or child* or cultur* or health or hous* or equipment or environment* or facility or facilities or operating or "pass through" or philanthrop* or planning or playground* or program* or project or projects or recreation* or senior* or service* or social or sport or "start* up" or start-up or support or technical or transportation or wellness or youth) N3 (endowment* or grant or grants or stipend*)) |
| S4 | AB ((grant* N3 fund*) or mini-grant* or minigrant* or "block grant*" or "seed grant*" or seed-grant* or (((endowment* or grant or grants or financial or funding or subsidy or subsidies or stipend*) N2 (application* or apply or applying or proposal* or submission* or submit* or requested or requesting)) or ((grant or grants or endowment* or subsidy or subsidies or stipend*) N2 (allocat* or awardee or awards or awarded or criteria or decision* or panel or recipient* or rule or rules or requirement* or regulation*)) or (funding N2 (academ* or communit* or decision* or research or project*)) or ((granted or granting or grants) N2 (cash or financ* or funds or money or monetary))) or ((apply or applying or application* or award* or grant or grants or granting) N3 (cash or funds or funding or financ* or money or monetary or income or subsidy or subsidies or stipend*)) or ((agency or agencies or charity or charities or civic or civilian* or department* or government* or group or groups or federal* or ministry* or ministries or ministerial or "not for profit" or non profit or project or projects or provinc* or public or region* or research or rural or state or territor* or tribe or tribal or urban) N3 (endowment* or grant or grants or stipend*))) |
| S3 | TI (grant or grants or (grant* N3 fund*) or endowment* or mini-grant* or minigrant* or "block grant*" or "seed grant*" or seed-grant* or ((endowment* or grant or grants or financial or funding or subsidy or subsidies or stipend*) N2 (application* or apply or applying or proposal* or submission* or submit* or requested or requesting)) or ((grant or grants or endowment* or subsidy or subsidies or stipend*) N2 (allocat* or awardee or awards or awarded or criteria or decision* or panel or recipient* or rule or rules or requirement* or regulation*)) or (funding N2 (academ* or communit* or decision* or research or project*)) or ((granted or granting or grants) N2 (cash or financ* or funds or money or monetary)) or ((apply or applying or application* or award* or grant or grants or granting) N3 (cash or funds or funding or financ* or money or monetary or income or subsidy or subsidies or stipend*)) or ((agency or agencies or charity or charities or civic or civilian* or department* or government* or group or groups or federal* or foundation* or ministry* or ministries or ministerial or national or "not for profit" or "non profit" or non-profit or project or projects or provinc* or public or region* or research or rural or state or territor* or tribe or tribal or urban) N3 (endowment* or grant or grants or stipend*))) OR KW (grant or grants or (grant* N3 fund*) or endowment* or mini-grant* or minigrant* or "block grant*" or "seed grant*" or seed-grant* or ((endowment* or grant or grants or financial or funding or subsidy or subsidies or stipend*) N2 (application* or apply or applying or proposal* or submission* or submit* or requested or requesting)) or ((grant or grants or endowment* or subsidy or subsidies or stipend*) N2 (allocat* or awardee or awards or awarded or criteria or decision* or panel or recipient* or rule or rules or requirement* or regulation*)) or (funding N2 (academ* or communit* or decision* or research or project*)) or ((granted or granting or grants) N2 (cash or financ* or funds or money or monetary)) or ((apply or applying or application* or award* or grant or grants or granting) N3 (cash or funds or funding or financ* or money or monetary or income or subsidy or subsidies or stipend*)) or ((agency or agencies or charity or charities or civic or civilian* or department* or foundation* or government* or group or groups or federal* or ministry* or ministries or ministerial or national or "not for profit" or "non profit" or non-profit or project or projects or provinc* or public or region* or research or rural or state or territor* or tribe or tribal or urban) N3 (endowment* or grant or grants or stipend*))) OR DE (grant or grants or (grant* N3 fund*) or endowment* or mini-grant* or minigrant* or "block grant*" or "seed grant*" or seed-grant* or ((endowment* or grant or grants or financial or funding or subsidy or subsidies or stipend*) N2 (application* or apply or applying or proposal* or submission* or submit* or requested or requesting)) or ((grant or grants or endowment* or subsidy or subsidies or stipend*) N2 (allocat* or awardee or awards or awarded or criteria or decision* or panel or recipient* or rule or rules or requirement* or regulation*)) or (funding N2 (academ* or communit* or decision* or research or project*)) or ((granted or granting or grants) N2 (cash or financ* or funds or money or monetary)) or ((apply or applying or application* or award* or grant or grants or granting) N3 (cash or funds or funding or financ* or money or monetary or income or subsidy or subsidies or stipend*)) or ((agency or agencies or charity or charities or civic or civilian* or foundation* or department* or government* or group or groups or federal* or national or ministry* or ministries or ministerial or national or "not for profit" or "non profit" or non-profit or project or projects or provinc* or public or region* or research or rural or state or territor* or tribe or tribal or urban) N3 (endowment* or grant or grants or stipend*))) OR SU (grant or grants or (grant* N3 fund*) or endowment* or mini-grant* or minigrant* or "block grant*" or "seed grant*" or seed-grant* or ((endowment* or grant or grants or financial or funding or subsidy or subsidies or stipend*) N2 (application* or apply or applying or proposal* or submission* or submit* or requested or requesting)) or ((grant or grants or endowment* or subsidy or subsidies or stipend*) N2 (allocat* or awardee or awards or awarded or criteria or decision* or panel or recipient* or rule or rules or requirement* or regulation*)) or (funding N1 (academ* or communit* or decision* or research or project*)) or ((granted or granting or grants) N2 (cash or financ* or funds or money or monetary)) or ((apply or applying or application* or award* or grant or grants or granting) N3 (cash or funds or funding or financ* or money or monetary or income or subsidy or subsidies or stipend*)) or ((agency or agencies or charity or charities or civic or civilian* or department* or government* or group or groups or federal* or foundation* or ministry* or ministries or ministerial or national or "not for profit" or "non profit" or non-profit or project or projects or provinc* or public or region* or research or rural or state or territor* or tribe or tribal or urban) N3 (endowment* or grant or grants or stipend*))) |
| S2 | TI ((granting or "granted by" or "endowment from") N3 (agency or agencies or body or bodies or foundation* or organisation* or organization*)) OR KW ((granting or "granted by" or "endowment from") N3 (agency or agencies or body or bodies or foundation* or organisation* or organization*)) OR SU ((granting or "granted by" or "endowment from") N3 (agency or agencies or body or bodies or foundation* or organisation* or organization*)) |
| S1 | DE "GRANTS (Money)" OR DE "GRANTS in aid (Public finance)" OR DE "DOMESTIC economic assistance" OR DE "SUBSIDIES" |

Database: Political Science Database <2008 to 2023 March 13>

| # | Query |
| --- | --- |
| 1 | (((MAINSUBJECT.EXACT("Federal aid to arts and humanities") OR MAINSUBJECT.EXACT("Community development block grants") OR MAINSUBJECT.EXACT("Government subsidies") OR MAINSUBJECT.EXACT("Government grants") OR MAINSUBJECT.EXACT("Federal aid to cities") OR MAINSUBJECT.EXACT("Federal aid to rural areas") OR MAINSUBJECT.EXACT("Federal aid to local governments")) OR subject(grant OR grants OR (grant* NEAR/3 fund*) OR endowment* OR mini-grant* OR minigrant* OR ("block grant" OR "block grants" OR "block grantsbeginning" OR "block grantthe") OR ("seed grant" OR "seed grants") OR seed-grant* OR ((endowment* OR grant OR grants OR financial OR funding OR subsidy OR subsidies OR stipend*) NEAR/2 (application* OR apply OR applying OR proposal* OR submission* OR submit* OR requested OR requesting)) OR ((grant OR grants OR endowment* OR subsidy OR subsidies OR stipend*) NEAR/2 (allocat* OR awardee OR awards OR awarded OR criteria OR decision* OR panel OR recipient* OR rule OR rules OR requirement* OR regulation*)) OR (funding NEAR/2 (academ* OR communit* OR decision* OR research OR project*)) OR ((granted OR granting OR grants) NEAR/2 (cash OR financ* OR funds OR money OR monetary)) OR ((apply OR applying OR application* OR award* OR grant OR grants OR granting) NEAR/3 (cash OR funds OR funding OR financ* OR money OR monetary OR income OR subsidy OR subsidies OR stipend*)) OR ((agency OR agencies OR charity OR charities OR civic OR civilian* OR department* OR government* OR group OR groups OR federal* OR ministry* OR ministries OR ministerial OR "not for profit" OR "non profit" OR non-profit OR project OR projects OR provinc* OR public OR region* OR research OR rural OR state OR territor* OR tribe OR tribal OR urban) NEAR/3 (endowment* OR grant OR grants OR stipend*))) OR title(grant OR grants OR (grant* NEAR/3 fund*) OR endowment* OR mini-grant* OR minigrant* OR ("block grant" OR "block grants" OR "block grantsbeginning" OR "block grantthe") OR ("seed grant" OR "seed grants") OR seed-grant* OR ((endowment* OR grant OR grants OR financial OR funding OR subsidy OR subsidies OR stipend*) NEAR/2 (application* OR apply OR applying OR proposal* OR submission* OR submit* OR requested OR requesting)) OR ((grant OR grants OR endowment* OR subsidy OR subsidies OR stipend*) NEAR/2 (allocat* OR awardee OR awards OR awarded OR criteria OR decision* OR panel OR recipient* OR rule OR rules OR requirement* OR regulation*)) OR (funding NEAR/2 (academ* OR communit* OR decision* OR research OR project*)) OR ((granted OR granting OR grants) NEAR/2 (cash OR financ* OR funds OR money OR monetary)) OR ((apply OR applying OR application* OR award* OR grant OR grants OR granting) NEAR/3 (cash OR funds OR funding OR financ* OR money OR monetary OR income OR subsidy OR subsidies OR stipend*)) OR ((agency OR agencies OR charity OR charities OR civic OR civilian* OR department* OR government* OR group OR groups OR federal* OR ministry* OR ministries OR ministerial OR "not for profit" OR "non profit" OR non-profit OR project OR projects OR provinc* OR public OR region* OR research OR rural OR state OR territor* OR tribe OR tribal OR urban) NEAR/3 (endowment* OR grant OR grants OR stipend*)))) AND (((MAINSUBJECT.EXACT("Community") OR  MAINSUBJECT.EXACT("Local government") OR MAINSUBJECT.EXACT("Community centers") OR MAINSUBJECT.EXACT("Neighborhoods") OR MAINSUBJECT.EXACT("Block clubs") OR MAINSUBJECT.EXACT("Megacities") OR MAINSUBJECT.EXACT("Municipal government") OR MAINSUBJECT.EXACT("Ghettos") OR MAINSUBJECT.EXACT("Federal aid to rural areas") OR MAINSUBJECT.EXACT("Rural health care") OR MAINSUBJECT.EXACT("Housing cooperatives") OR MAINSUBJECT.EXACT("Urban planning") OR MAINSUBJECT.EXACT("Gated communities") OR MAINSUBJECT.EXACT("Homeowners associations") OR MAINSUBJECT.EXACT("Master planned communities") OR MAINSUBJECT.EXACT("Rural areas") OR MAINSUBJECT.EXACT("Urban areas") OR MAINSUBJECT.EXACT("Community organizations") OR MAINSUBJECT.EXACT("Cities") OR MAINSUBJECT.EXACT("Urban development") OR MAINSUBJECT.EXACT("Community action") OR MAINSUBJECT.EXACT("Towns") OR MAINSUBJECT.EXACT("Neighborhood watch programs") OR MAINSUBJECT.EXACT("Suburban areas") OR MAINSUBJECT.EXACT("Community relations") OR MAINSUBJECT.EXACT("Rural development") OR MAINSUBJECT.EXACT("Municipalities") OR MAINSUBJECT.EXACT("Metropolitan areas") OR MAINSUBJECT.EXACT("Community development") OR MAINSUBJECT.EXACT("Urban renewal")) OR ((MAINSUBJECT.EXACT("Multidisciplinary teams") OR MAINSUBJECT.EXACT("Organizations") OR MAINSUBJECT.EXACT("Teams") OR MAINSUBJECT.EXACT("Support groups") OR MAINSUBJECT.EXACT("Community centers") OR MAINSUBJECT.EXACT("Clubs") OR MAINSUBJECT.EXACT("Cultural centers") OR MAINSUBJECT.EXACT("Hispanic serving institutions") OR MAINSUBJECT.EXACT("Nonprofit organizations") OR MAINSUBJECT.EXACT("Charities") OR MAINSUBJECT.EXACT("Boards of trustees") OR MAINSUBJECT.EXACT("Housing cooperatives") OR MAINSUBJECT.EXACT("Alumni associations") OR MAINSUBJECT.EXACT("Cultural organizations") OR MAINSUBJECT.EXACT("Fraternal organizations") OR MAINSUBJECT.EXACT("Historical organizations") OR MAINSUBJECT.EXACT("Youth organizations") OR MAINSUBJECT.EXACT("Arts management") OR MAINSUBJECT.EXACT("Homeowners associations") OR MAINSUBJECT.EXACT("Community supported agriculture") OR MAINSUBJECT.EXACT("Student organizations") OR MAINSUBJECT.EXACT("Religious organizations") OR MAINSUBJECT.EXACT("Religious congregations") OR MAINSUBJECT.EXACT("Veterans organizations") OR MAINSUBJECT.EXACT("Agricultural cooperatives") OR MAINSUBJECT.EXACT("Cooperatives") OR MAINSUBJECT.EXACT("Nongovernmental organizations NGOs") OR MAINSUBJECT.EXACT("Collaboration") OR MAINSUBJECT.EXACT("Learned societies") OR MAINSUBJECT.EXACT("Foundations")) OR (MAINSUBJECT.EXACT("Teams") OR MAINSUBJECT.EXACT("Groups") OR MAINSUBJECT.EXACT("Teamwork") OR MAINSUBJECT.EXACT("Clubs") OR MAINSUBJECT.EXACT("North American Indian agencies") OR MAINSUBJECT.EXACT("Social classes") OR MAINSUBJECT.EXACT("Health clubs") OR MAINSUBJECT.EXACT("Sports & recreation clubs") OR MAINSUBJECT.EXACT("Councils") OR MAINSUBJECT.EXACT("Minority & ethnic groups") OR MAINSUBJECT.EXACT("Refugees") OR MAINSUBJECT.EXACT("Youth volunteers") OR MAINSUBJECT.EXACT("Student organizations") OR MAINSUBJECT.EXACT("Sports facilities") OR MAINSUBJECT.EXACT("Day care centers") OR MAINSUBJECT.EXACT("Community organizations") OR MAINSUBJECT.EXACT("Cultural identity") OR MAINSUBJECT.EXACT("Volunteers") OR MAINSUBJECT.EXACT("Native peoples") OR MAINSUBJECT.EXACT("Tribal society") OR MAINSUBJECT.EXACT("Cooperatives") OR MAINSUBJECT.EXACT("School councils") OR MAINSUBJECT.EXACT("Disabled people") OR MAINSUBJECT.EXACT("Performing arts centers") OR MAINSUBJECT.EXACT("Adult day care") OR MAINSUBJECT.EXACT("Pedestrians") OR MAINSUBJECT.EXACT("Senior citizen centers"))) OR (MAINSUBJECT.EXACT("Multidisciplinary teams") OR MAINSUBJECT.EXACT("College campuses") OR MAINSUBJECT.EXACT("Campuses") OR MAINSUBJECT.EXACT("Social structure") OR MAINSUBJECT.EXACT("Social support") OR MAINSUBJECT.EXACT("Agricultural cooperatives") OR MAINSUBJECT.EXACT("Cooperatives") OR MAINSUBJECT.EXACT("Ethnicity") OR MAINSUBJECT.EXACT("Low income groups") OR MAINSUBJECT.EXACT("Parent teacher groups") OR MAINSUBJECT.EXACT("Grass roots movement") OR MAINSUBJECT.EXACT("Agrarian society") OR MAINSUBJECT.EXACT("Communities of practice") OR MAINSUBJECT.EXACT("Working groups") OR MAINSUBJECT.EXACT("Tribal society") OR MAINSUBJECT.EXACT("Collaboration"))) OR (title(alliance OR charity OR charities OR club OR clubs OR community OR communities OR congregation* OR facility OR group* OR guild* OR league* OR organisation* OR organization* OR society OR societies OR groups* OR foundation* OR league*) OR mainsubject(alliance OR charity OR charities OR club OR clubs OR community OR communities OR congregation* OR facility OR group* OR guild* OR league* OR organisation* OR organization* OR society OR societies OR groups* OR foundation* OR league*)))) AND (stype.exact("Scholarly Journals") AND pd(20080101-20230313)) |
| 2 | (MAINSUBJECT.EXACT("Social networks") OR MAINSUBJECT.EXACT("Organizations") OR MAINSUBJECT.EXACT("Organizational structure") OR MAINSUBJECT.EXACT("Organizational change") OR MAINSUBJECT.EXACT("Organization development") OR MAINSUBJECT.EXACT("Social network analysis") OR MAINSUBJECT.EXACT("Group dynamics") OR MAINSUBJECT.EXACT("Organization theory") OR MAINSUBJECT.EXACT("Social structure") OR MAINSUBJECT.EXACT("Organizational behavior")) AND ((MAINSUBJECT.EXACT("Federal aid to arts and humanities") OR MAINSUBJECT.EXACT("Community development block grants") OR MAINSUBJECT.EXACT("Government subsidies") OR MAINSUBJECT.EXACT("Government grants") OR MAINSUBJECT.EXACT("Federal aid to cities") OR MAINSUBJECT.EXACT("Federal aid to rural areas") OR MAINSUBJECT.EXACT("Federal aid to local governments")) OR subject(grant OR grants OR (grant* NEAR/3 fund*) OR endowment* OR mini-grant* OR minigrant* OR ("block grant" OR "block grants" OR "block grantsbeginning" OR "block grantthe") OR ("seed grant" OR "seed grants") OR seed-grant* OR ((endowment* OR grant OR grants OR financial OR funding OR subsidy OR subsidies OR stipend*) NEAR/2 (application* OR apply OR applying OR proposal* OR submission* OR submit* OR requested OR requesting)) OR ((grant OR grants OR endowment* OR subsidy OR subsidies OR stipend*) NEAR/2 (allocat* OR awardee OR awards OR awarded OR criteria OR decision* OR panel OR recipient* OR rule OR rules OR requirement* OR regulation*)) OR (funding NEAR/2 (academ* OR communit* OR decision* OR research OR project*)) OR ((granted OR granting OR grants) NEAR/2 (cash OR financ* OR funds OR money OR monetary)) OR ((apply OR applying OR application* OR award* OR grant OR grants OR granting) NEAR/3 (cash OR funds OR funding OR financ* OR money OR monetary OR income OR subsidy OR subsidies OR stipend*)) OR ((agency OR agencies OR charity OR charities OR civic OR civilian* OR department* OR government* OR group OR groups OR federal* OR ministry* OR ministries OR ministerial OR "not for profit" OR "non profit" OR non-profit OR project OR projects OR provinc* OR public OR region* OR research OR rural OR state OR territor* OR tribe OR tribal OR urban) NEAR/3 (endowment* OR grant OR grants OR stipend*))) OR title(grant OR grants OR (grant* NEAR/3 fund*) OR endowment* OR mini-grant* OR minigrant* OR ("block grant" OR "block grants" OR "block grantsbeginning" OR "block grantthe") OR ("seed grant" OR "seed grants") OR seed-grant* OR ((endowment* OR grant OR grants OR financial OR funding OR subsidy OR subsidies OR stipend*) NEAR/2 (application* OR apply OR applying OR proposal* OR submission* OR submit* OR requested OR requesting)) OR ((grant OR grants OR endowment* OR subsidy OR subsidies OR stipend*) NEAR/2 (allocat* OR awardee OR awards OR awarded OR criteria OR decision* OR panel OR recipient* OR rule OR rules OR requirement* OR regulation*)) OR (funding NEAR/2 (academ* OR communit* OR decision* OR research OR project*)) OR ((granted OR granting OR grants) NEAR/2 (cash OR financ* OR funds OR money OR monetary)) OR ((apply OR applying OR application* OR award* OR grant OR grants OR granting) NEAR/3 (cash OR funds OR funding OR financ* OR money OR monetary OR income OR subsidy OR subsidies OR stipend*)) OR ((agency OR agencies OR charity OR charities OR civic OR civilian* OR department* OR government* OR group OR groups OR federal* OR ministry* OR ministries OR ministerial OR "not for profit" OR "non profit" OR non-profit OR project OR projects OR provinc* OR public OR region* OR research OR rural OR state OR territor* OR tribe OR tribal OR urban) NEAR/3 (endowment* OR grant OR grants OR stipend*)))) AND (stype.exact("Scholarly Journals") AND pd(20080101-20230313)) |
