# Supplementary material for: Mobilizing community-driven health promotion through community granting programs: a rapid systematic review
Source: BMC Public Health. 2024 Apr 1;24:932. doi: 10.1186/s12889-024-18443-8 (PMC10983705; doi:10.1186/s12889-024-18443-8)
Supplement: Supplementary file 2 — Supplementary Material 2. [file 12889_2024_18443_MOESM2_ESM.docx]

**Appendix 2: Quality Assessments of Included Studies**

**Table A2a: Studies assessed using JBI Checklist for Quasi-Experimental Studies**

| Study | JBI Checklist for Quasi-Experimental Studies | | | | | | | | | | |
| --- | --- | --- | --- | --- | --- | --- | --- | --- | --- | --- | --- |
|  | 1. Clear cause and effect | 2. Similar participants in comparisons | 3. Participants received similar treatment | 4. Control group | 5. Multiple outcome measurements | 6. Follow up complete or differences described | 7. Outcomes measured in same way | 8. Reliable outcome measurement | 9. Appropriate statistical analysis | Overall score* | Rating^†^ |
| Mayberry, 2009 (52) | Yes | Yes | Yes | No | Yes | Yes | Yes | Yes | Yes | 8 | High |
| Sharpe, 2015 (51) | Yes | No | No | No | No | Yes | Yes | No | No | 3 | Low |

*Only items that received a “Yes” were counted toward the overall score.

^†^Ratings were based on the total criteria met by the study (1-3 Low; 4-6 Moderate, 7-9 High).

**Table A2b: Studies assessed using JBI Checklist for Analytical Cross Sectional Studies**

| Study | JBI Checklist for Analytical Cross Sectional Studies | | | | | | | | | |
| --- | --- | --- | --- | --- | --- | --- | --- | --- | --- | --- |
|  | 1. Clear inclusion criteria | 2. Subjects and setting described | 3. Valid and reliable exposure measurement | 4. Objective, standard condition measurement | 5. Confounding factors identified | 6. Strategies for confounding factors | 7. Valid and reliable outcome measurement | 8. Appropriate statistical analysis | Overall score* | Rating^†^ |
| Grossman, 2019 (53) | No | Yes | Yes | Yes | Yes | No | Yes | Yes | 6 | Moderate |

*Only items that received a “Yes” were counted toward the overall score.

^†^Ratings were based on the total criteria met by the study (1-3 Low; 4-6 Moderate, 7-8 High).

**Table A2c: Studies assessed using JBI Checklist for Qualitative Research**

| Study |  | JBI Checklist for Qualitative Research | | | | | | | | | | | |
| --- | --- | --- | --- | --- | --- | --- | --- | --- | --- | --- | --- | --- | --- |
|  | 1. Congruity with research methods | | 2. Congruity with research question | 3. Congruity with data collection methods | 4. Congruity with data analysis | 5. Congruity with results interpretation | 6. Statement locating the researcher theoretically | 7. Researcher influence addressed | 8. Participants adequately represented | 9. Ethical approval | 10. Conclusions flow from data | Overall score* | Rating^†^ |
| Abildso, 2019 (47) | Yes | | Yes | Yes | Yes | Yes | No | No | Yes | Yes | Yes | 8 | High |
| Coombe, 2023 (45) | No | | Yes | Yes | No | Yes | No | No | Yes | Yes | Yes | 6 | Moderate |
| Goodman, 2017 (40) | No | | Yes | Yes | Yes | Yes | No | No | Yes | No | Yes | 6 | Moderate |
| Hickey, 2015 (46) | Yes | | Yes | Yes | Yes | Yes | No | No | Yes | No | Yes | 7 | Moderate |
| Honeycutt, 2012 (44) | Yes | | Yes | Yes | Yes | Yes | No | No | Yes | Yes | Yes | 8 | High |
| Nieves, 2020 (48) | Yes | | Yes | Yes | Yes | Yes | No | No | Yes | Yes | Yes | 8 | High |
| Pearson, 2020 (41) | No | | Yes | Yes | Yes | Yes | No | No | Yes | No | Yes | 6 | Moderate |
| Ramanathan, 2018 (39) | No | | Yes | Yes | Yes | Yes | No | No | Yes | Yes | Yes | 7 | Moderate |
| Schmidt, 2009 (49) | No | | Yes | Yes | No | Yes | No | No | Yes | No | Yes | 5 | Moderate |
| Soares, 2014 (43) | No | | Yes | Yes | Yes | Yes | No | No | No | No | Yes | 5 | Moderate |
| Tamminen, 2014 (90) | No | | Yes | Yes | Yes | Yes | No | No | Yes | Yes | Yes | 7 | Moderate |
| Thompson, 2010 (50) | No | | Yes | Yes | Yes | Yes | No | Yes | Yes | No | Yes | 7 | Moderate |
| Vanderpool, 2011 (42) | Yes | | Yes | Yes | Yes | Yes | No | No | Yes | Yes | Yes | 8 | High |
| Wyatt, 2011 (69) | No | | Yes | Yes | No | Yes | No | No | No | No | Yes | 4 | Low |

*Only items that received a “Yes” were counted toward the overall score.

^†^Ratings were based on the total criteria met by the study (1-4 Low; 5-7 Moderate, 8-10 High).
